# Structure and dynamics of liquid water from *ab initio* simulations: adding Minnesota density functionals to Jacob's ladder†

**DOI:** 10.1039/d3sc05828j

**Published:** 2024-02-15

**Authors:** Justin Villard, Martin P. Bircher, Ursula Rothlisberger

**Affiliations:** a Laboratory of Computational Chemistry and Biochemistry, Institute of Chemical Sciences and Engineering, École Polytechnique Fédérale de Lausanne (EPFL) Lausanne CH-1015 Switzerland ursula.roethlisberger@epfl.ch; b Computational and Soft Matter Physics, Universität Wien Wien A-1090 Austria

## Abstract

The accurate representation of the structural and dynamical properties of water is essential for simulating the unique behavior of this ubiquitous solvent. Here we assess the current status of describing liquid water using *ab initio* molecular dynamics, with a special focus on the performance of all the later generation Minnesota functionals. Findings are contextualized within the current knowledge on DFT for describing bulk water under ambient conditions and compared to experimental data. We find that, contrary to the prevalent idea that local and semilocal functionals overstructure water and underestimate dynamical properties, M06-L, revM06-L, and M11-L understructure water, while MN12-L and MN15-L overdistance water molecules due to weak cohesive effects. This can be attributed to a weakening of the hydrogen bond network, which leads to dynamical fingerprints that are over fast. While most of the hybrid Minnesota functionals (M06, M08-HX, M08-SO, M11, MN12-SX, and MN15) also yield understructured water, their dynamical properties generally improve over their semilocal counterparts. It emerges that exact exchange is a crucial component for accurately describing hydrogen bonds, which ultimately leads to corrections in both the dynamical and structural properties. However, an excessive amount of exact exchange strengthens hydrogen bonds and causes overstructuring and slow dynamics (M06-HF). As a compromise, M06-2X is the best performing Minnesota functional for water, and its D3 corrected variant shows very good structural agreement. From previous studies considering nuclear quantum effects (NQEs), the hybrid revPBE0-D3, and the rung-5 RPA (RPA@PBE) have been identified as the only two approximations that closely agree with experiments. Our results suggest that the M06-2X(-D3) functionals have the potential to further improve the reproduction of experimental properties when incorporating NQEs through path integral approaches. This work provides further proof that accurate modeling of water interactions requires the inclusion of both exact exchange and balanced (non-local) correlation, highlighting the need for higher rungs on Jacob's ladder to achieve predictive simulations of complex biological systems in aqueous environments.

## Abstract 2

This study incorporates Minnesota density functionals into the current knowledge of describing the structural and dynamical properties of liquid water through *ab initio* molecular dynamics, with M06-2X(-D3) functionals showing the most promise.

## Graphical abstract

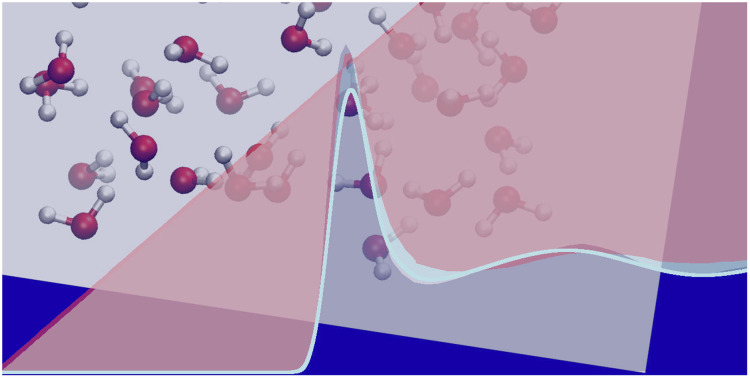

## 1 Introduction

Liquid water is a ubiquitous and essential component of life, playing a critical role in a wide variety of chemical and biological processes.^1–4^ A comprehensive understanding of water at the atomic scale is vital for advancing research in diverse domains such as aqueous chemistry,^5–8^ biochemistry,^9,10^ atmospheric science,^11,12^ and environmental engineering.^13,14^ Furthermore, unraveling the intricate behavior of water molecules enables deeper insights into solvation dynamics,^15,16^ water–materials interactions,^17,18^ protein folding,^19–22^ enzymatic reactions,^23,24^ and the properties of biological membranes,^25,26^ ultimately contributing to the development of innovative technologies and therapeutics. Deceivingly simply at first sight, it is well known that liquid water shows anomalous properties that have been extensively observed and documented like the density anomaly,^2,27,28^ high heat capacity,^10,25,29^ high boiling and melting points,^3,30^ high surface tension,^31,32^ high dielectric constant,^33,34^ and high viscosity.^35,36^ Despite substantial advances in the understanding of the behavior of water, the origins of these anomalies are not yet entirely elucidated neither by experiments nor theory, although it has been widely recognized that the structural characteristics of the hydrogen bond network under thermal fluctuations play a pivotal role for these unique features.^4,37–41^

Significant challenges exist in conclusively capturing atomic-scale phenomena in water through experiments like NMR,^42–52^ IR,^53^ X-ray^54,55^ or neutron^55–58^ spectroscopy for which measurement interpretations often rely on theoretical models. Although a variety of computationally efficient and relatively accurate empirical force fields have been developed,^59–63^ such as those from the TIPnP family,^64,65^ those remain intrinsically incapable of describing bond breaking in chemical reactions. Therefore, the quantitative understanding of condensed phase water, in particular its reactivity, and role as a universal solvent can only fully emerge from the development of accurate *ab initio* molecular dynamics (AIMD) simulations.^66–68^ These simulations need to faithfully represent both electronic reorganization and nuclear quantum effects (NQEs) associated with hydrogen bonding but, at present, such a comprehensive predictive quantum picture at ambient conditions remains quite elusive. In addition, the cost of most accurate wavefunction-based approaches such as post-Hartree–Fock^69,70^ (*e.g.*, MP2 ^71^ or RPA^72–75^), coupled cluster (CCSD(T)),^76,77^ or configuration interaction (CI)^78,79^ hinders their potential application across the entire water phase diagram.

Balancing accuracy and computational feasibility, Kohn–Sham (KS)^80^ density functional theory (DFT)^81^ has become the go-to quantum-chemical method for time propagation of molecular systems and computation of statistical averages when combined with molecular dynamics (MD) or Monte-Carlo (MC) engines.^63,82^ Although the ground-state energy and electron density are formally exact within DFT, their universal mapping remains unknown, necessitating the use of approximations in the KS formalism. In this approach, many-body interactions are accounted for and incorporated into the approximate exchange–correlation (XC) functional.

Over the past several decades, hundreds of XC functionals have been developed with the aim to capture all relevant physics and achieve chemical accuracy over a broad range of molecules, materials, and organometallic systems.^83^ To classify the growing number of functionals, John P. Perdew and Karla Schmidt proposed a hierarchy called Jacob's ladder,^84^ which organizes functionals based on their complexity. The ladder consists of five rungs: (1) Local Density Approximations (LDA) depend only on the electron density at a given point in space and offer computational efficiency but often lack accuracy.^85,86^ (2) Generalized Gradient Approximations (GGA) functionals incorporate the (local) electron density and its gradient.^87–89^ (3) Meta-GGA functionals account for the electron density, its gradient, and the kinetic energy density.^90–94^ (4) Hybrid functionals mix a portion of exact exchange‡‡As emphasized by a reviewer, the nomenclature of exact exchange refers herein to the single-determinant exchange evaluated from the Fock integral of orbitals coming from hybrid functionals. This term should therefore not be confused with the exact KS exchange, obtained by evaluating the Fock form with the KS determinant, in association with the exact and local KS potential. from Hartree–Fock (HF) theory with XC terms from DFT,^95–99^ and (5) double-hybrid and RPA-based functionals (rung-5), the highest rung on the ladder, combine a hybrid functional with post-HF correlation corrections, *e.g.*, within second-order perturbation theory (MP2)^71,100–106^ or non-local correlation based on the random phase approximation (RPA).^107–111^ As one moves up the ladder, the functionals globally tend to provide better descriptions of electronic interactions and improve the overall predictive accuracy.^84,112–116^ This is primarily attributed to the introduction of orbital-dependent terms at the meta-GGA rung 3 level, which enables the XC potential to become non-local in what is known as generalized KS-DFT, in contrast to traditional KS-DFT, where the XC potential remains local. However, this comes at the price of an increasingly higher computational cost: for instance, the cost of hybrids is roughly two orders of magnitude the one of GGA functionals.^115,117–119^

While DFT with refined density functional approximations has demonstrated impressive success in the examination of structures, properties, and reactivities for a wide range of molecules and materials, the prominent challenge persists in identifying the appropriate XC functional for a specific problem, as the performance of a functional can vary significantly depending on the system under study.^120^ For liquid water, no local (LDA) or semilocal (GGA, meta-GGA) DFT simulation has yet achieved a conclusive replication of experimental observations, covering both structural and dynamical properties simultaneously. For example, it was established that most of the GGA functionals, like PBE^89^ and BLYP,^87,88^ provide a more pronounced dip after the first peak of the oxygen–oxygen pair correlation function, thus an overstructured description of water, and dynamical figures that are too slow, therefore not completely remedying the glassy behavior observed with the LDA.^121–125^ Furthermore, GGA and (even) hybrid levels can underestimate the equilibrium density of liquid water, leading to the incorrect prediction that ice sinks in water.^41,126^

DFT approximations encounter difficulties when describing condensed water due to the intricate nature of concurrent competing interactions that are involved in covalent bonds, hydrogen bonds, and van der Waals (vdW) forces. Hydrogen bonds, though one order of magnitude weaker than intramolecular O–H covalent bonds, remain locally strong and directionally attractive. Another order of magnitude weaker, vdW dispersion forces play a non-negligible role at larger distances, with an attractive and isotropic character.^127^ The interplay between varying interaction strengths, length scales, and directionalities makes water a highly sensitive test system for the design and assessment of XC functionals. Indeed, even slight imprecision in the XC description is likely to disrupt the complex balance of interactions, ultimately impacting the H-bond network that is responsible for many of water's properties.^125,128^

While local and semilocal functionals fail to capture intermediate to long-range vdW forces,^125^ AIMD simulations have demonstrated that GGAs enhanced with vdW representations typically lead to a softer structure of bulk water where peak maxima and minima in the radial distribution functions are less pronounced, accompanied by increased mobility that aligns more closely with experimental measurements.^124^ This improvement is achieved by incorporating dispersion-corrected atom-centered potentials (DCACPs),^124,129,130^ empirical dispersion corrections (*e.g.*, Grimme's D2 ^131^ and D3 ^132^), or non-local correlation terms (*e.g.*, (r)VV10,^133–135^ vdW-DF,^136^ TS-vdW^127,137^). However, the performance of such corrections relies on the original GGA to which the combination may not always improve, or may even deteriorate properties.^124,138^ Other studies pointed out the necessity of including a fraction of exact exchange, thus resorting to rung-4 hybrids, to effectively describe hydrogen bonding but without reaching a perfect agreement with the experiment.^125,139–143^

Altogether, attaining a reliable description of the structural and dynamical properties of liquid water through lower rung (1–3) DFT models remains an issue. The goal of this work is consequently to contribute further understanding to this endeavor by incorporating the popular Minnesota density functionals^92,144–153^ into the array of approximations tested on water at ambient conditions. While having demonstrated success for molecular systems, previous investigations of the performance of Minnesota functionals on condensed water are, to our knowledge, limited to the work of Del Ben *et al.* who ran MC simulations on water with the M06-L-D3, M06-D3, and M06-2X-D3 functionals,^154^ and the work of Pestana *et al.* that focuses on MD with M06-L-D3.^143^ Our work thus fills a gap in the evaluation of the performance of DFT functionals for liquid water. Gaining insights from the performance of various functionals not only helps demystify their promise and limitations for water, but also on a wider range of systems exhibiting a similar delicate balance of interactions such as *e.g.*, in large biomolecules,^155,156^ heterogeneous catalysts,^157,158^ aqueous solutions^7,159,160^ and molecules on surfaces.^161^ For this reason, we have made an effort, albeit not exhaustive, to compile in this document previously calculated quantities from DFT-based MC and AIMD. Our aim is to establish a common ground for comparing various studies found in the literature and confront them with experimental measurements.

Information on higher-rung approximations, such as double-hybrids, is limited in this assessment due to their exorbitant computational overhead and infrequent implementation in MD software packages.^154^ The substantial cost of hybrid functionals also poses a significant challenge for obtaining extensive results in MD simulations,^140,162^ in particular in the context of plane wave based approaches. To tackle this issue, the emergence of machine learning (ML)-based interaction models has shown the potential to attain a similar level of accuracy at a fraction of the cost.^163–165^ Nevertheless, the effectiveness of such ML potentials primarily depends on their reliability across the entire phase (configurational) space sampled during MD (MC) simulations.

Hereafter, we present structural properties (in terms of radial distribution functions, coordination numbers, density, number of H-bonds and angular distributions) and dynamical characteristics (quantified *via* diffusion coefficients and rotational correlation times) obtained with AIMD and all the later generation Minnesota functionals. Those include some of the most employed meta-GGAs and hybrid meta-GGAs in computational chemistry.^115,166,167^ Meta-GGAs are investigated with Car–Parrinello MD (CPMD), while the much more computationally expensive hybrid meta-GGAs have been run with Born–Oppenheimer MD (BOMD), thanks to the crucial acceleration of a recent ML-aided multiple time step scheme that preserves the target DFT level description by construction.^119,168,169^ Both CPMD and BOMD employ classical propagation of nuclei; however, capturing a comprehensive picture of water including nuclear quantum effects (NQEs) requires more sophisticated and considerably costlier (approximately two orders of magnitude^117^) *ab initio* path integral MD (PIMD) approaches.^63,170^ Alternatively, NQEs can be qualitatively evaluated based on very recent studies that employ DFT/ML-based PIMD methods.^40,163,164^ This allows an identification of the most promising XC functionals worth further investigation in conjunction with quantum nuclei.

In this regard, this article provides benchmarks for the widely-used Minnesota density functionals in simulating liquid water, and places them in the context of existing knowledge of other DFT approximations as well as experimental measurements. Anticipating our results, it turns out that M06-2X is not only the best of all Minnesota functionals tested, but also rivals the currently most promising functionals reported overall, especially if combined with dispersion corrections (M06-2X-D3), and considered in the light of NQEs.

## 2 Theory and methods

### 2.1 Minnesota density functionals

Since 2005, the Minnesota theoretical chemistry group led by Donald Truhlar has focused on the development of post-GGA functionals capable of capturing the chemistry of main group elements as well as transition metals including activation barriers as well as non-covalent interactions. The excellent “across-the-board” performance of these functionals has made them one of the most widely used XC approximations in computational chemistry.^7,115,166,167^ The Minnesota functionals are semi-empirical in nature, with functional forms that have been fitted against extensive datasets of reference absolute and relative energies, as well as eventual structures and lattice constants. For brevity's sake, Table 1 provides a summary of the XC approximations studied in this work, along with a global overview of their functional components. Interested readers are referred to the corresponding references for more technical and mathematical details.

**Table 1:** Overview of some characteristic features of Minnesota density functionals, in terms of *E*_xc_ = (*X*/100)*E*^HF^_x_ + (1 − *X*/100)*E*^DFT^_x_ + *E*^DFT^_c_. *X* is the percentage of exact exchange in the functional. *E*^DFT^_x_ and *E*^DFT^_c_ depicts the origins of the functional form for the exchange (*e.g.*, exchange energy density, correction factors) and the correlation (*e.g.*, correlation energy density, gradient correction).^a^ Also listed are the number of fitted parameters # as well as the satisfaction (✓) or not (×) of the uniform electron gas (UEG) limit

Functional	Class^b^	*X* [%]	*E* ^DFT^_x_	*E* ^DFT^_c_	#	UEG	Ref.
**Meta-GGA**							
M06-L	L meta-GGA	0	M05 + VSXC	M05 + VSXC	34	✓	92
revM06-L	L meta-GGA	0	M05 + VSXC	M05 + VSXC	31	× only	144
M11-L	RSL meta-GGA	0	SR/LR: LSDA(PBE + RPBE)	LSDA + PBE	44	✓	145
MN12-L	L meta-NGA	0	N12	N12 + (LSDA + PBE)	58	×	146
MN15-L	L meta-NGA	0	N12	N12 + (LSDA + PBE)	58	×	147

**Hybrid meta-GGA**							
M06	GH meta-GGA	27	M05 + VSXC	M05 + VSXC	33	✓	148
M06-HF	GH meta-GGA	100	M05 + VSXC	M05 + VSXC	32	✓	149
M06-2X	GH meta-GGA	54	M05	M05 + VSXC	29	✓	148
M08-HX	GH meta-GGA	52.23	LSDA(PBE + RPBE)	LSDA + PBE	47	✓	150
M08-SO	GH meta-GGA	56.79	LSDA(PBE + RPBE)	LSDA + PBE	44	✓	150
M11	RSH meta-GGA	42.8–100	SR: LSDA(PBE + RPBE)	LSDA + PBE	40	✓	151
MN12-SX	RSH meta-NGA	25–0	N12	N12 + (LSDA + PBE)	58	×	152
MN15	GH meta-NGA	44	N12	N12 + (LSDA + PBE)	59	×	153

a SR stands for short-range, LR for long-range. LSDA is the local spin density approximation,^171,172^ PBE the Perdew, Burke, Ernzerhof functional,^89^ RPBE the secondly revised PBE functional,^173^ and N12 Truhlar's non-separable density gradient functional.^174^

b L stands for local, RSL for range-separated local, GH for global hybrid, RSH for range-separated hybrid and NGA for non-separable gradient approximation.

The generation of the 2006 functionals was ingeniously crafted by merging the characteristics of the earlier M05 ^175^ and VSXC^176^ functionals (in turn designed from modifications of the PBE and LSDA functionals for the exchange). These include M06, a versatile hybrid meta-functional that boasts consistent accuracy for main group thermochemistry, barrier heights, medium-range correlation energies, and transition metals. M06-2X, another hybrid meta-GGA, excels in main group chemistry and barrier heights, accurately predicts valence and Rydberg electronic excitation energies, and π–π stacking interactions, while its performance falters in the realm of transition metals. M06-L, a local functional devoid of Hartree–Fock exchange, was skillfully tailored as a cost-effective choice for numerous demanding applications associated with extensive systems. It excels for transition metals, yet its accuracy for barrier heights does not match that of M06 and M06-2X. Finally, M06-HF was designed primarily for spectroscopy, demonstrating good performance for valence, Rydberg, and charge transfer excited states with little compromise on ground-state accuracy. An important point to note is that M06-2X and M06-HF that differ in the amount of exact exchange (54 *vs.* 100%) share the same training set, which was expanded with transition metals with respect to the one used for the parameterization of the M06 functional. revM06-L, on the other hand, was developed later using an even larger database and additional smoothness restraints to ensure better numerical stability, smoother potential energy curves, and overall improved accuracy compared to M06-L.

The next generation functionals M08-HX and M08-SO resulted from exploring a more flexible functional form, with different formal constraints; while M08-SO respects the exact gradient expansion for slowly varying density up to the second order (SO) and the uniform electron gas (UEG) limit, M08-HX only respects the latter. Both functionals of the M08 generation were found to modestly improve on M06-2X for main-group thermochemistry, kinetics, and non-covalent interactions. The even more recent M11, on the other hand, is a range-separated version^177^ of the M08 functionals, with the same correlation component. The percentage of exact exchange of 100% at large inter-electronic distance reduces to 42.8% at short range. The second-order density gradient expansion is also correct by construction in M11, and good across-the-board accuracy was shown thanks to the use of a further extended training set. A bit later, the M11-L functional was designed as the local analogue of M11, mainly for cost-efficiency and improved accuracy for multi-reference systems. M11-L replaces the exact exchange by a long-range meta-GGA exchange functional, that has different spatial extent and parameters than the exchange at short range.

In 2012, a new functional form called N12 was developed that pushes the limits of local functionals, providing simultaneous accuracy on energetic and structural properties of both solids and molecules.^174^ Unlike traditional GGAs, the N12 functional is a non-separable approximation (NGA) between the density and its (reduced) gradient that embodies both exchange and correlation effects, and can be seen as a generalization of the dual-range M11-L. By adding a dependence on the kinetic energy density, and the M08/M11 correlation term, Peverati and Truhlar designed the MN12-L meta-non-separable gradient approximation to obtain even broader accuracy with a local functional. With the inclusion of 25% of short-range exact exchange (that is screened at large distances), the MN12-SX functional yields better results than MN12-L for most chemical properties, and is notably more successful in calculating semiconductor band gaps.^152^ Finally, re-optimization of MN12-L using a larger training database and additional smoothness restraints on the functional form resulted in the most recent MN15-L local meta-NGA functional. This latter shows better performance for transition metals and is generally recommended over MN12-L.^147^ Its hybrid version, called MN15, was trained using a combination of single-reference chemical data (barrier heights), as well as diverse multi-reference transition-metal bond energies and atomic excitation energies that are challenging to describe with KS-DFT. As a result, it provides broad accuracy for both multi-reference and single-reference systems, and at the same time has demonstrated outstanding performance in describing noncovalent interactions.^153^ Note that we include NGAs in the category of GGAs in the rest of the text for simplicity's sake. Also, we emphasize that explicit dispersion corrections to the Minnesota density functionals were not considered in the present work.

### 2.2 Simulations

AIMD simulations were carried out using the CPMD code^178^ with PBE Troullier–Martins norm-conserving pseudopotentials.^179^ We observed that this universal choice has a negligible effect when comparing the optimization of a water molecule with Minnesota functionals and PBE pseudopotentials/plane waves to all-electron Gaussian basis set calculations. When convergence limits and integration grids are tight, and basis sets large, the difference between all-electron and pseudopotential/plane-wave calculations becomes negligible, a reassuring conclusion that has also been observed recently on Hartree–Fock and correlation energies.^180^ The plane-wave wavefunction cutoff energy was set to 80 Ry for all systems. We used a finer integration mesh with a density cutoff energy set to 640 Ry (dual of 8) to ensure proper convergence of the Minnesota functionals with planes waves,^167^ therefore affecting the usual computational cost by a factor of 2. The convergence threshold for the DIIS^181^ wavefunction optimization was set to 10^−6^ a.u. on the residual gradient on occupied orbitals, except for the M06 functional that is harder to converge to such a low criterion and for which 5 × 10^−6^ a.u. was used instead.

#### 2.2.1 Meta-GGA functionals

For the simulations with meta-GGAs, systems use a cubic 12.445^3^ Å^3^ periodic box of 64 water molecules corresponding to a density of ∼1 g cm^−3^, simulated *via* Car–Parrinello MD. The wavefunction fictitious mass is chosen to be 800 a.u. and all hydrogens were assigned the mass of deuterium to increase the integration time steps.

A first equilibration phase was performed for each functional. Starting with a pre-equilibrated structure at the classical level, systems were first heated up to 400 K with velocity rescaling for about 1 ps until reaching a stable average temperature. Then, systems were cooled down to 330 K during another picosecond, and the temperature was again decreased more slowly to 300 K during a time interval of about half a picosecond.

After the first initial equilibration, systems were further thermalized with a Nosé–Hoover thermostat on the ions at 300 K for several picoseconds with a coupling frequency of 1500 cm^−1^ before finally switching to the *NVE* ensemble for the production runs for at least 10 ps. Configurations were saved every 50 steps for analysis. More information about the lengths of the trajectories, time steps and energy conservation are reported in Table S1 of the ESI.†

#### 2.2.2 Hybrid meta-GGA functionals

Due to their high computational cost, the AIMD simulations with hybrid functionals were performed with a smaller cubic box of dimensions 9.939^3^ Å^3^ containing 32 water molecules. A multiple time step (MTS) scheme^169,182,183^ was used to further accelerate the simulations, with an inner time step of *δt* = 15 a.u. and an outer time step of Δ*t* = *n*·*δt*, where the time step ratio *n* is chosen to maintain sufficient energy conservation. At inner time steps, fast force components are given by a delta-ML model that predicts PBE0 forces based on the LDA (***F***^inner^ = ***F***^LDA^ + Δ***F***^PBE0-LDA^_ML_), while total forces are corrected at the outer time step with their slow components (***F***^outer^ = ***F***^Minnesota^ − ***F***^inner^) to fully recover the higher-level Minnesota forces.^119,168^ In this approach, ML serves only as a low-level surrogate operating on shorter timescales without impacting the target DFT level. Note that the inner PBE0 level does not need to match the outer Minnesota level entirely, but should be close enough so that their difference slowly varies in time and dynamically decouples from fast force components. Ultimately, the Minnesota level is recovered at larger physical time steps by construction, ensuring that the structural and dynamical properties are not affected,^119,184^ unlike in ML-potential MD.

The OQML^185,186^ kernel method is used to infer force differences Δ***F***^PBE0-LDA^_ML_ from the aSLATM^187^ representations of chemical environments. The training set for the OQML model was generated by running PBE0 trajectories on condensed water and small water clusters. Both energies and forces were used in the training. The model demonstrated an out-of-sample mean absolute error of around 0.3 kcal (mol^−1^ Å^−1^) on |Δ***F***^PBE0-LDA^_ML_|, as well as a mean absolute error of 0.7° on force directions, based on a test set of 4800 atomic forces.

Starting from a PBE0 pre-equilibrated configuration, all systems were first thermalized in the *NVT* ensemble with the ML-MTS acceleration method and a Nosé–Hoover thermostat with a coupling frequency of 1500 cm^−1^ at 300 K for at least 5 ps. After this initial equilibration process, *NVE* runs were conducted during the production phase, sampling configurations for at least 6 ps. The lengths of the trajectories, time step ratios, and energy conservation are reported in the ESI (Table S1†).

### 2.3 Analysis

Here, we provide information on how the properties were calculated from AIMD trajectories. As the production runs were conducted in the *NVE* ensemble, the average temperature of each simulation slightly differs. To ensure comparability, care was taken to renormalize the properties either by considering temperature or box volume differences.

We note that the average structural properties are similar in the *NVT* and *NVE* ensembles.^184^ Additionally, the replacement of hydrogen atoms with deuterium has little effect on structural properties when the ionic motion is treated classically.^121,139^ However, the use of deuterated water can affect dynamical properties, such as the diffusion coefficient. Therefore, it is important to rely on heavy water data when validating D_2_O simulations against experimental results.

#### 2.3.1 Radial distribution functions and coordination number

Radial distribution functions (RDFs) were computed using the VMD software,^188^ accounting for periodic boundary conditions and a bin width of 0.01 Å. The RDFs are then smoothed by interpolation for integration and visualization purposes with negligible differences when compared to the original statistical averages. The coordination numbers *n*_OO_ of water molecules is obtained as the oxygen–oxygen (O–O) coordination number resulting from the integral of the O–O RDF *g*_OO_:^54^1
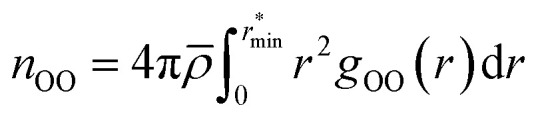
where *ρ̄* is the molecular number density. For consistency with experimental reference data, the value of 
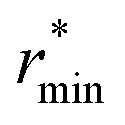
 is set as the position of the first minimum in the actual integrand *r*^2^*g*_OO_(*r*), rather than the first minimum of *g*_OO_(*r*). For comparison, we also report the coordination number *n̄*_OO_ calculated up to the first minimum of *g*_OO_(*r*) in the ESI (Table S3†).

#### 2.3.2 Density of liquid water

The equilibrium density predicted by the Minnesota functionals is estimated by scanning over volume changes around trajectory snapshots.^122^ For each snapshot, total energies are calculated at scaled values of the lattice constant. The intramolecular coordinates are held fixed while the positions of the centers of mass of the water molecules are rescaled to scan over volume reductions and expansions. The equilibrium volume and density are determined by calculating the minimum of the interpolated energy values, at a given snapshot. 30 snapshots were used, each separated by 0.2 ps, to obtain a representative set of configurations. The density is calculated from the average equilibrium volume over all snapshots. Given that the basis set size varies with the volume of the box in plane wave basis sets, a larger wavefunction cutoff energy of 200 Ry was used to ensure reliable energy differences from these calculations.

#### 2.3.3 H-bond number and angular distributions

**Fig. 1:**
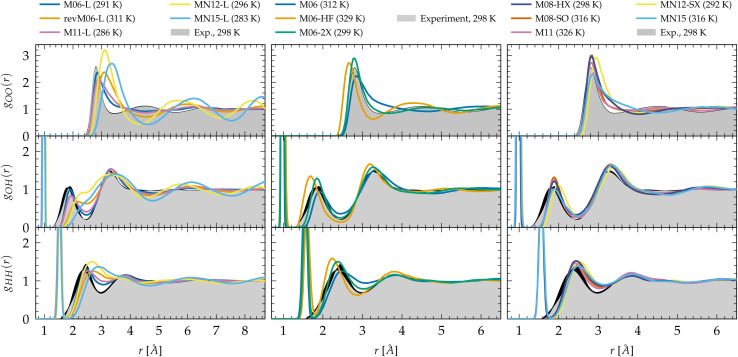
Oxygen–oxygen (*g*_OO_), oxygen–hydrogen (*g*_OH_) and hydrogen–hydrogen (*g*_HH_) radial distribution functions of liquid water predicted by Minnesota density functionals. The experimental reference for *g*_OO_ comes from X-ray diffraction^54,55^ interpolated at 298 K ^197^ and joint X-ray/neutron diffraction experiments were used for *g*_OH_ and *g*_HH_.^58^ Black areas represent experimental uncertainties.

The number of hydrogen bonds is evaluated from geometrical criteria following ref. 124 and 189: a first function is defined to reflect the probability of having an H-bond formed between molecules *i* and *j* based on the distance 
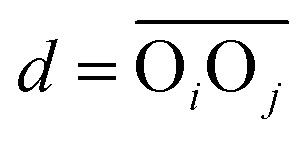
 between the respective oxygen atoms. According to the experimental O–O RDF (*cf.*Fig. 1), neighboring oxygen atoms in the first coordination shell are located at distances between 2.4 and 3.4 Å. To reflect this, a smoothed rectangle function is defined by the following polynomial approximation2
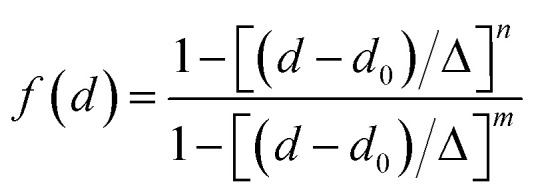
where *d*_0_ = 2.8 Å characterizes the center of the rectangle and corresponds to the first peak of the O–O RDF. *Δ* = 0.45 Å defines the rectangle width that covers the range of the first coordination shell, and *n* = 10 and *m* = 16 are reasonable smoothing parameters that have no significant impact on the results if varied towards close but even values. A second metric involves the distance 
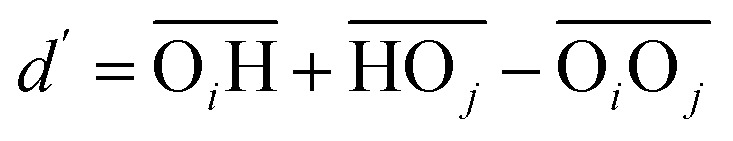
 (with the donor oxygen O_*i*_ and its covalently-bound hydrogen H, and the distance 
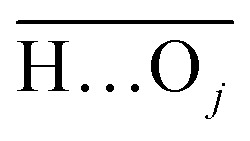
 to the corresponding acceptor oxygen O_*j*_). This latter metric increases in particular when the donor-hydrogen direction is tilted. As *d*′ increases, the probability of hydrogen bonding gradually decreases to zero. This is described by the second function 
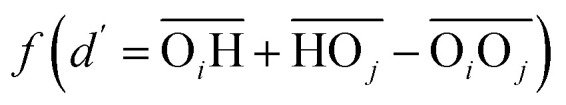
 (eqn (2)) with *d*_0_ = 0, *Δ* = 0.4 Å, *n* = 4 and *m* = 8. With such parameters, *f*(*d*′) equals 1 at 0 Å, and rapidly decays to 0 when the argument exceeds 0.5 Å. Here again, the results do not differ significantly even if the smoothing parameters *n* and *m* are chosen somewhat differently. With this, an H-bond is finally counted if the product of the two functions exceeds 0.5, and not otherwise. The presence or absence of an H-bond is facilitated because the product of these analytical functions is predominantly either close to 0 or to 1. In practice, it is defined whether H is covalently bound to either molecule *i* or *j* in order to ensure the correct counting with periodic boundary conditions. We have observed, like others,^189^ that this counting is qualitatively comparable to conventional criteria that involve both the 
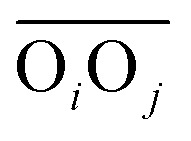
 distance and the angle between the O_*i*_O_*j*_ and O_*i*_H directions.^139^

To compute the H-bond angular distributions, we took into account all the molecules present in the first coordination shell of the reference molecule, *i.e.* we restricted our analysis to angles for which the donor–acceptor distance is less than 3.4 Å, and the hydrogen-acceptor distance is less than 2.5 Å, based on the experimental RDFs.^54,55^

#### 2.3.4 Diffusion coefficient

The self-diffusion coefficient *D*_L_ is calculated from the Einstein relation3
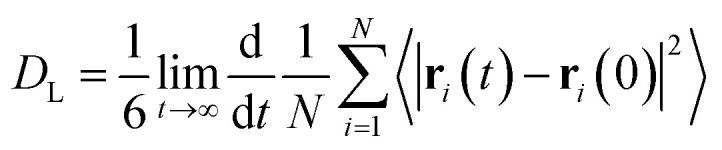
where *N* is the number of water molecules, **r**_*i*_(*t*) the position of each oxygen atom *i* at time *t*, and the brackets indicate an average over the *NVE* ensemble. Improved statistics were gathered across multiple lag times and time origins according to the default parameters of the Diffusion Coefficient Tool plugin^190^ for VMD^188^ to finally obtain *D*_L_ from the average slope of the mean-squared displacement (MSD).

Since *D*_L_ is calculated from the simulation of a *L*^3^ cubic water box, finite size effects are corrected *via*^191^4
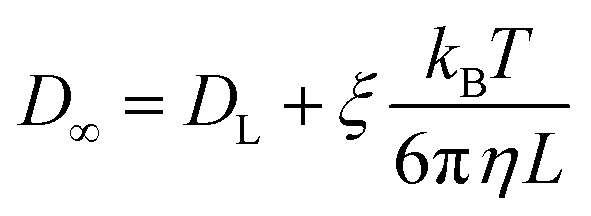
where *D*_∞_ is the infinite-size limit, *ξ* = 2.837297, *k*_B_ the Boltzmann constant, and *η* the shear viscosity of the fluid at average temperature *T*. The viscosity *η* predicted by each functional approximation is generally not known, and relying on the experimental value^192^ was observed not to significantly affect the rescaling of *D*_L_ to *D*_∞_.^191^ In this regard, theoretical viscosities were computationally derived for SCAN and optB88-vdW.^40^ We observed negligible deviations in *D*_∞_ when calculated using either experimental or theoretical viscosities (Table S4†). However, if a functional is too overstructured, it may predict a larger viscosity, leading to an overestimation of *D*_∞_ when calculated with the experimental (lower) viscosity. Another reliable approach to compare with experiment is to rescale the experimental coefficients *D*^exp^_∞_ back to *D*^exp^_L_, which is the hypothetical experimental value for a box of size *L*.^193^

#### 2.3.5 Orientational correlation times

In addition to the translational motion, the rotational time scale of the water molecules is determined by analyzing the orientational auto-correlation function:5
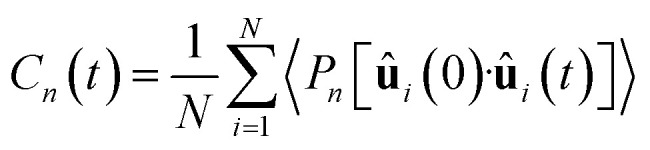
where *P*_*n*_ is the Legendre polynomial of order *n* = 1, 2 and **û**_*i*_ is the molecular unit vector along either the OH covalent bonds, the HH intramolecular direction, or the direction of the dipole moment *μ*. The rotational correlation times *τ*_1,2_ were determined by fitting the curves *C*_*n*=1,2_(*t*) with the function *A*e^−*t*/*τ*_1,2_^ in the exponential regime following the initial subpicosecond decay, which is due to the librational motion of the water molecules.^194,195^ These relaxation times have been found to be less affected by finite-size effects compared to the self-diffusion coefficient,^196,197^ and are of interest because they can be measured experimentally using techniques such as NMR^2,42,43,45,46,50,52,198,199^ or IR^200,201^ spectroscopy.

## 3 Results and discussion

### 3.1 Structural properties

#### 3.1.1 Radial distribution functions

The radial pair distribution functions (RDFs in terms of *g*_OO_, *g*_OH_, *g*_HH_) provide structural information as modelled by the different Minnesota functionals. In Fig. 1, we compare respectively the O–O, O–H and H–H RDFs to experimental references. The left panel reports the results of meta-GGAs. Clearly, the O–O RDFs indicate that M06-L and M11-L are understructured, with first *g*_OO_ minima that are too high and too far. These two functionals also behave alike when it comes to the O–H and H–H distributions that are slightly understructured compared to experiment. Although yielding similar RDFs, it is interesting to recall that M06-L and M11-L do not share the same exchange and correlation functional forms as well as training data, and that M11-L is range-separated (Table 1). However, both fulfill the UEG limit. For the remaining functionals where this constraint is lifted (revM06-L, MN12-L, MN15-L), the O–H and H–H RDFs move even further away from the experiment and no longer capture the hydrogen-bond network as shown by the smearing out of the second peak in the *g*_OH_ distributions. The revM06-L functional has the same XC form as M06-L, but differs in the imposed constraints and training data. In contrast to the others, the MN12-L and MN15-L non-separable functionals result in an overstructured *g*_OO_ but again lack exactness in the intermolecular distances^202^ with typical shifts in the location of the first minimum up to 1 Å. MN15-L was designed from a re-optimization of MN12-L using a larger database and additional smoothness restraints on the functional form. Therefore, the RDF similarities between M06-L and M11-L (different forms, different training data) and differences between M06-L/revM06-L and MN12-L/MN15-L (same form, different constraints, different training data) would advocate for a larger sensitivity of semi-empirical meta-GGAs to exact constraints rather than training data. Consistent with this, the additional smoothness restraints in revM06-L (*versus* M06-L), and MN15-L (*versus* MN12-L) seem to reduce the packing of water molecules and shift the first peak of the O–O RDF to larger distance. Overall, no local (*i.e.*, non-hybrid) Minnesota functional is providing an accurate reproduction of the structure of liquid water, mainly due to failures in the description at intermediate and long-range intermolecular distances.

The hybrid functionals of the M06 family are shown in the center panel of Fig. 1. Interestingly, M06 predicts RDFs that are very similar to its M06-L sister. M06-L therefore appears as a good local functional fit for the 27%-hybrid M06, but both fail at reproducing the intermolecular structure of water at long range.^155^ In contrast, the increase of exact exchange to 100% in M06-HF noticeably over-accentuates the structure and shifts the first and second *g*_OO_ peaks to too short intermolecular distances. This increased cohesive effect that was missing for the local functionals is also observed in the *g*_OH_ and *g*_HH_ RDFs.

As a compromise between M06 and M06-HF, M06-2X, with 54% of exact exchange, remarkably improves the agreement of the RDFs with experiments. Despite the first minimum of *g*_OO_ being a bit right-shifted by ∼0.3 Å, M06-2X shows better peak positions and an improved second coordination shell according to the second peak in *g*_OO_. As observed, the agreement with experimental data is not a trivial matter, as the structure of water is the result of the complex interplay between covalent bonds, hydrogen bonds, and vdW interactions. Many-body effects among hydrogen-bonded water molecules can be observed in the first peak of *g*_OO_ and the second peak of *g*_OH_. The region between the first and second peaks of *g*_OO_ mainly consists of non-hydrogen-bonded water molecules that occupy the intershell space between the hydrogen-bonded neighbors. The increased number of water molecules in these intershell regions can partly be attributed to the attractive, non-directional vdW interactions.^41,143^ Therefore, achieving a balance between exact exchange and vdW dispersion at an intermediate length scale is essential for accurately reproducing the densely packed and disordered structure in the intershell regions. As demonstrated by the RDFs, M06-2X captures these correlations with the highest accuracy and is thus capable of describing both hydrogen bonding and dispersion effects. M06-2X was specifically designed with the absence of transition metals in its training set, and focuses on the description of the electron correlation of the main group elements which could be one of the reasons why it performs so well on water compared to M06 for which transition metals were included. M06-HF lacks an adequate amount of correlation to counterbalance the full exact exchange: the second coordination shell has a higher population of water molecules that are not sufficiently drawn out to the intershell region by vdW forces.

The newer generation Minnesota hybrid functionals do not improve the structural description any further (Fig. 1, right panel). While possessing nearly the same amount of exact exchange as M06-2X, the new functional form introduced in M08-HX (52%) and M08-SO (57%) does not outperform M06-2X. MN12-SX is both range-separated and non-separable, with 25% of exact exchange at short range that decreases to 0% at long range. This functional has the lowest proportion of exact exchange. Notably, it is also the one where the first *g*_OO_ and the second *g*_OH_ peaks are shifted to the right, *i.e.* to longer intermolecular distances, presumably due to an elongation (weakening) of the intermolecular hydrogen bonds, or a lack of vdW cohesive forces^202^ (the analysis of the dynamical properties in Section 3.2 confirms the second hypothesis). In general, it is observed that the inclusion of a fraction of exact exchange leads to clearly visible improvements in the *g*_OH_ and *g*_HH_ RDFs over local functionals, and the addition of an appropriate amount of exact exchange can also enhance the agreement for *g*_OO_. This is particularly the case for M11, MN12-SX and MN15 that improve the second peak of *g*_OH_ significantly over M11-L, MN12-L and MN15-L, respectively. Moreover, although not perfect, these functionals clearly improve the position and shape of the first *g*_OO_ peak compared to their local counterparts. Consequently, this emphasizes the crucial importance of exact exchange in accurately describing the hydrogen bond network in general, supporting the notion that hybrid functionals and higher rungs of Jacob's ladder are indeed the most accurate approaches for depicting complex interactions with KS-DFT.

**Fig. 2:**
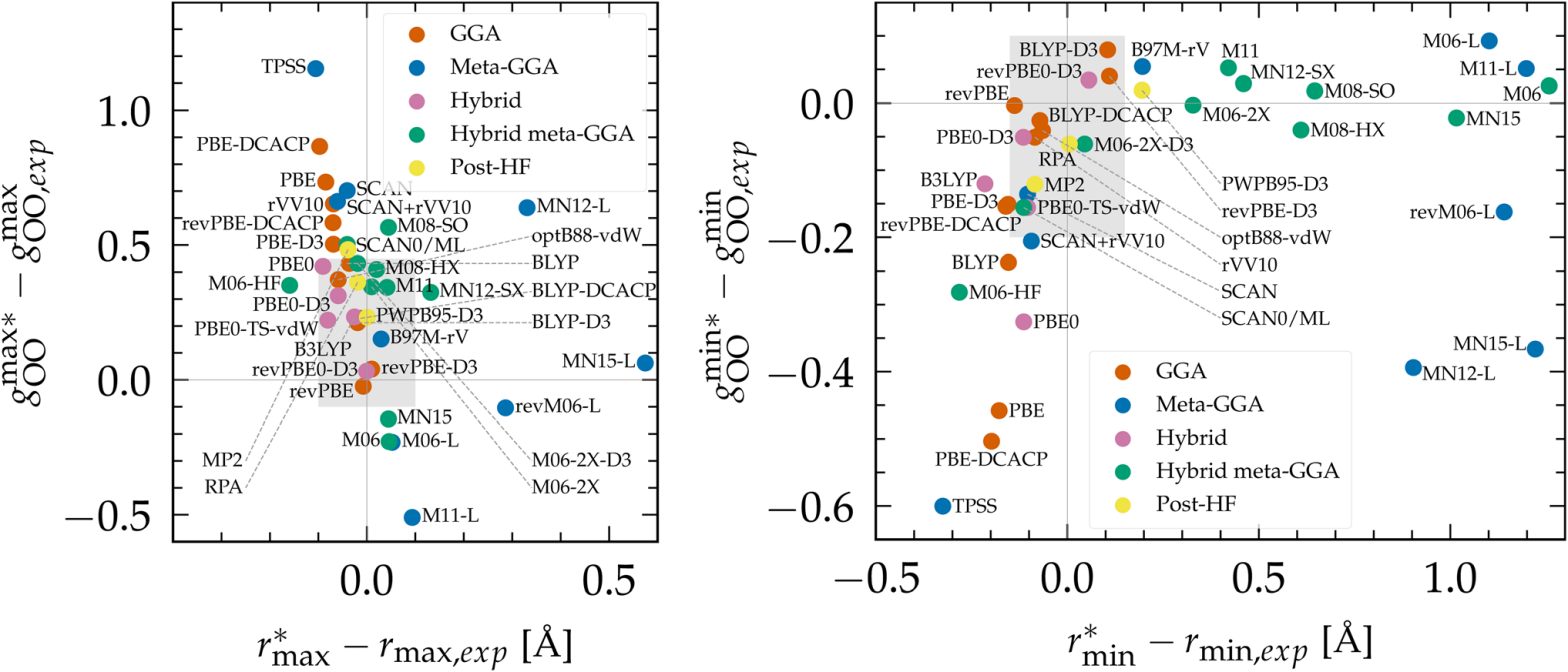
(Left) Difference between the rescaled position 
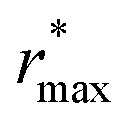
 and height 
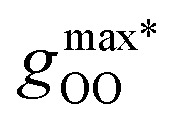
 of the first *g*_OO_ maximum and the experimental values at 298 K. (Right) Difference between the rescaled position 
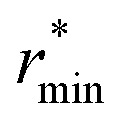
 and height 
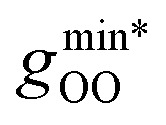
 of the first *g*_OO_ minimum and the experimental values at 298 K. Values for non-Minnesota functionals were extracted from ref. 40, 117, 124, 138, 140, 143, 154, 162, 164 and 165 and are reported in Table S2 of the ESI.† Rescaled values were obtained through empirical interpolation of experimental data.^55^ The grey areas represent a visual estimate of the potential deviations resulting from the neglect of nuclear quantum effects as well as statistical and experimental uncertainties (*cf.* Section 3.1.2).

To evaluate the performance of Minnesota functionals in the broader context of DFT approximations, we compiled a comprehensive dataset from the literature (Table S2 of the ESI†). As various functionals were employed at different temperatures, the position and height of the first *g*_OO_ peak, as well as the first *g*_OO_ minimum, were rescaled to a common reference point at 298 K based on empirical interpolations fitted to experimental data (Fig. S2†). The differences between simulated and experimental values are depicted in Fig. 2. As can be seen, KS-DFT coupled to a classical propagation of the nuclei have the tendency to generally overestimate the height of the first peak and underestimate the first minimum, resulting in an overstructured prediction of liquid water. This is a well-known result for approximations lacking vdW interactions, such as purely local GGAs.^124,143,154^ Although dispersion corrections generally represent a step in the right direction, *i.e.* a less overstructured RDF, their effect depends on the specific functional and correction employed. For instance, BLYP is improved when supplemented with either D3 and DCACP corrections, while PBE is only improved with the D3 correction and deteriorates with DCACP (which was attributed to the presence of artificial dispersion effects in PBE^124^). Notably, the rVV10 non-local functional is also overstructured. In summary, BLYP-DCACP and revPBE-D3 are the best GGA functionals reported so far for the structure of liquid water.

The importance of a sensitive tweaking of non-local dispersion effects is likely the primary reason why meta-GGA functionals do not exhibit improvement over the best dispersion-corrected GGAs. Compared to all functionals, the local Minnesota are the worst, as they cause substantial right-shifting and broadening of the first *g*_OO_ peak. In contrast, the SCAN functional appears to capture the intermediate-ranged vdW interactions which seem to help locating the *g*_OO_ maximum and minimum at good distance,^41^ but SCAN remains overstructured. The difference in results between SCAN and its augmentation with the rVV10 non-local correlation functional (SCAN + rVV10) is negligible.^138^ However, this add-on does help the B97M-rV functional to become the best meta-GGA reported.

Based on the available data, hybrids provide a good approximation of the first maximum of *g*_OO_, which is consistent with our previous observation that the inclusion of exact exchange in Minnesota functionals improves the accuracy of both the position and height of the first peak. This can be attributed to the fraction of exact exchange that mitigates the self-interaction error in local and semilocal XC functionals, which has been correlated with an artificial strengthening of the H_2_O tetrahedral structure and the delocalization of protons.^164^ Although PBE0 still yields overly structured water, its D3 and TS-vdW variants provide better agreement with experimental data. The most accurate hybrid functional appears to be revPBE0-D3, which is also the best approximation over all functionals for which data on water has been reported (*vide infra*).

Moving on to hybrid meta-GGAs, indications of the performance of SCAN0, the hybridized version of SCAN, has been obtained from simulations based on a deep neural network potential (SCAN0/ML) which indicate that SCAN0 is still overstructured. With the exception of M06-HF with 100% exact exchange, the hybrid Minnesota functionals are generally accurate in predicting the height of the first minimum, but they fail to accurately predict its position (Fig. 2, right). However, Del Ben *et al.* discovered that M06-2X, which appears to be the best performing Minnesota functional for water overall, further improves when coupled with the D3 correction.^154^ In general, the first minimum 
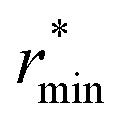
 is shifted to a smaller intermolecular distance when there is either an excessive amount of exchange (M06-HF) or when the correlation effects overestimate vdW interactions. This highlights the remarkable sensitivity between (exact) exchange and correlation, both of which tend to compress or augment the first coordination peak instead of having compensatory effects. Achieving an accurate description of liquid water with DFT therefore requires finding the correct balance between these two quantum effects. This quest has motivated the refinement of XC functionals, where the occupied and virtual KS orbitals contribute to non-local correlation just as the occupied orbitals contribute to the non-local exact exchange. From Fig. 2, the RPA, which consists of exact exchange plus the RPA correlation, appears as the most promising rung-5 (post-HF) DFT approach in this direction, *e.g.*, yielding very good structural properties outperforming MP2.^154,165^

According to this comprehensive comparison, the most accurate functionals for describing the structure of water with classical nuclei are: revPBE-D3, BLYP-DCACP (GGA), B97M-rV (meta-GGA), revPBE0-D3 (hybrid), M06-2X-D3 (hybrid meta-GGA) and the RPA (rung-5).

#### 3.1.2 Nuclear quantum effects

The low mass of the hydrogen atom makes nuclear quantum effects (NQEs) significant when simulating water properties.^154,184,203^ For example, tunneling effects can affect the formation and breaking of hydrogen bonds and influence the dynamics.^139^ The results presented in this work (Fig. 2) should therefore be interpreted in light of the fact that NQEs are absent in CPMD or BOMD dynamics with a classical propagation of nuclei. As an illustration, taking into account NQEs with revPBE-D3 revealed that its good agreement with water properties using classical nuclei is due to a fortuitous cancellation of errors, where the neglect of exact exchange compensates for the neglect of quantum nuclei.^117,143,204^ Advanced path integral molecular dynamics (PIMD) methods are necessary for quantum-mechanical treatment of nuclei, particularly when comparing high-level electronic structure calculations with experimental results.^63,203,205^ However, this comes at the cost of approximately two orders of magnitude more computational expense than simulations where the nuclei are treated classically. As a result, it has been common practice to mimic NQEs by performing classical (nuclei) MD at elevated temperatures increased by around 30 K.^41,162^ While this ad hoc technique was found to provide reasonable accuracy for RDFs, it often fails to correctly reproduce the dynamical properties that become too fast compared to proper NQEs.^41,117,163,184^ Alternatively, recent advances have enabled the acceleration of PIMD dynamics, especially with the help of ML potentials that infer DFT energies and forces at a much reduced cost.^40,163–165^

As expected, the general trend observed in PIMD simulations is that NQEs tend to soften the structure of liquid water: for BLYP,^203^ SCAN/ML,^40,163^ PBE0-D3,^154^ SCAN0/ML,^164^ RPA/ML^165^ and MP2/ML,^206^ less structured RDFs were found when including NQEs. For other functionals like SCAN,^184^ B97M-rV^117^ and revPBE0-D3,^117,204^ O–O RDFs remain almost unchanged, while the O–H and H–H RDFs become less structured. O–H and H–H RDFs are also less structured for BLYP-D3 ^184^ and revPBE-D3,^204^ that however have a slight decrease in the O–O first minimum (by ∼0.1) when adding NQEs, with no impact on the first maximum. However, overall NQEs seem to have a marginal influence on the positions of the maxima and minima of the distribution functions. Hence, classical RDFs tend to be either too structured or very similar to their quantum analogues. This is in agreement with experimental isotope studies between heavy and light water that also showed that NQEs soften the structure of liquid water.^40,162,207^ Hence, NQEs partially explain why in Fig. 2 most DFT functionals tend to overstructure water compared to the experimental results. The gray areas plotted in this figure account for possible deviations due to the neglect of NQEs. These are based on the previously-cited PIMD references, potential discrepancies between experimental measurements,^54,55,58^ and the variance of the rescaling procedure to 298 K. These areas therefore enclose the most promising XC functionals to be predictive when including NQEs.

According to previous studies, the best functionals tested so far for describing the atomic structure of water with NQEs are: revPBE-D3 ^204^ (GGA), B97M-rV^117^ (meta-GGA), revPBE0-D3 ^117,204^ (hybrid), SCAN0/ML^164^ (hybrid meta-GGA) and RPA/ML^165^ (rung-5). However, good agreement with experiment was only obtained for revPBE0-D3 and the RPA (from insights with ML potentials). The other levels of theory still overstructure water when considering NQEs, except for B97M-rV that remains understructured. Therefore, from Fig. 2, other XC approximations that would be worth investigating with PIMD simulations would be: optB88-vdW, BLYP-DCACP (GGA), PBE0-D3(TS-vdW) (hybrid) and M06-2X(-D3) (hybrid meta-GGA). Running PIMD calculations with rung-5 XCs like the RPA, without the aid of ML, would be of interest but their cost currently prevents such endeavors.

Finally, we note that NQEs influence the balance between covalent and hydrogen bond interactions. Indeed, PIMD simulations showed that NQEs broaden the covalent peak of the O–H RDF, meaning that more fluctuations occur for the hydrogen atoms, accompanied by a weakening of the covalent bonds. In turn, such a delocalization of the protons seems to strengthen the hydrogen bond network by forming statistically more H-bond interactions, which slows down dynamical properties.^117,154,163,184,204^ Thus, counterintuitively, the disordering due to NQEs smoothes out the structure of water by destabilizing molecules in the intershell region of the O–O RDF, while simultaneously reducing diffusion and rotational times due to stronger hydrogen bonds. Such findings are crucial in order to analyze dynamical properties in light of NQEs (Section 3.2).

#### 3.1.3 Coordination number

**Fig. 3:**
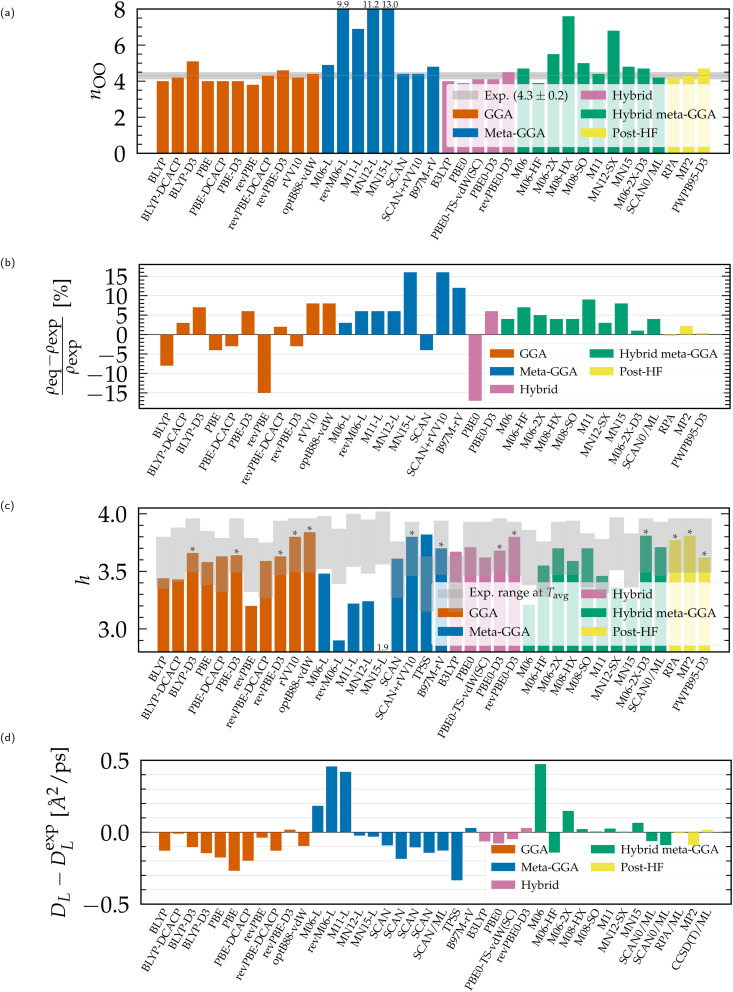
Structural and dynamical properties of liquid water from DFT-based *ab initio* simulations, compared to experimental values.^51,54,55,192,208,209^ (a) Coordination number, (b) equilibrium density, (c) average number of H-bonds per water molecule (*upper bound from the integration of *g*_OH_ instead of geometric criteria), (d) finite-size diffusion coefficient. Results for non-Minnesota functionals were extracted from ref. 40, 41, 117, 124, 138, 140, 143, 154, 162–165, 184 and 197 and reported in Tables S3 and S4 of the ESI.†

The coordination number *n*_OO_ predicted by each functional is plotted in Fig. 3a. Experimentally, Skinner *et al.* showed that the O–O coordination number of the liquid state has a value of 4.3 and is independent of temperature,^54,55^ while previous works reported values between 4 and 5.^39,54,55,57,124^ In addition, negligible changes were observed from AIMD and force field simulations at different temperatures,^124^ supporting that deviations of *n*_OO_ directly relate to the quality of the intermolecular interactions as described by the functionals. As seen, a majority of them is in agreement with the tetrahedral configuration of nearest-neighbor water molecules.^41,54^ However, the fact that the O–O RDF does not reach zero after the first peak makes it challenging to determine the first coordination shell unambiguously. This difficulty makes *n*_OO_ strongly dependent on the distance cutoff selected for the integration of the RDF: in most cases, *n*_OO_ is slightly underestimated because the O–O RDF tends to be overstructured in the absence of NQEs. On the other hand, the smoothening due to the addition of dispersion corrections makes the theoretical predictions agree more closely with experiments (*e.g.*, BLYP-DCACP, revPBE-DCACP, M06-2X-D3). The lack of accuracy of the Minnesota meta-GGAs is further exemplified by their extended first coordination shell that includes an unphysical number of water molecules. Although still understructured, this is partly corrected for some hybrid functionals such as M06, M06-2X(-D3), M08-SO, M11, and MN15.

#### 3.1.4 Density of liquid water

As illustrated in Fig. 3b, GGA functionals tend to underestimate the equilibrium density, which is rectified by adding dispersion corrections. The incorrect prediction that ice sinks in water with local DFT is mainly due to the absence of dispersion in plain GGA functionals.^41,125^ However, meta-GGA functionals such as SCAN have been shown to correct this issue.^41^ The PBE0 hybrid functional faces challenges in achieving the right balance between covalent, hydrogen bonds and vdW forces. It significantly underestimates the density, but this can be improved with the D3 correction. For all other meta-GGAs, hybrids, hybrid meta-GGAs and rung-5/post-HF, the density is higher than the experimental value. Overall, vdW interactions increase the density because of their attractive and isotropic nature at intermediate and long range. This increases the population of molecules in the intershell regions of the O–O distribution function, *i.e.* between the coordination shells, and acts as additional cohesive force in the condensed phase. Consistent with their structural differences (Fig. 1), the increase in the amount of exact exchange in the M06, M06-2X and M06-HF also correlates with a rise of the density. On the other hand, in a counteracting manner, the delocalization and disordering effects due to NQEs can be expected to reduce the density, explaining why DFT densities with classical nuclei are usually overestimated.

#### 3.1.5 H-bond number and angular distributions

From their atomic composition, water molecules in ice ideally arrange in a tetrahedral coordination made of four hydrogen bonds per molecule. In liquid water, entropic effects bend, stretch, break and reform hydrogen bonds such that the average number of H-bonds per molecule is slightly less than 4 (∼3.8) at near ambient conditions.^41,143^ This average number *h* is plotted in Fig. 3c, where the gray boxes indicate the estimated discrepancy among various experimental methods at the simulated temperature.^208,209^ Our observations, and those of others,^139^ suggest that the computation of *h* is relatively insensitive to changes in temperature, with a small deviation of approximately 0.1 for every 10 K increase.

**Fig. 4:**
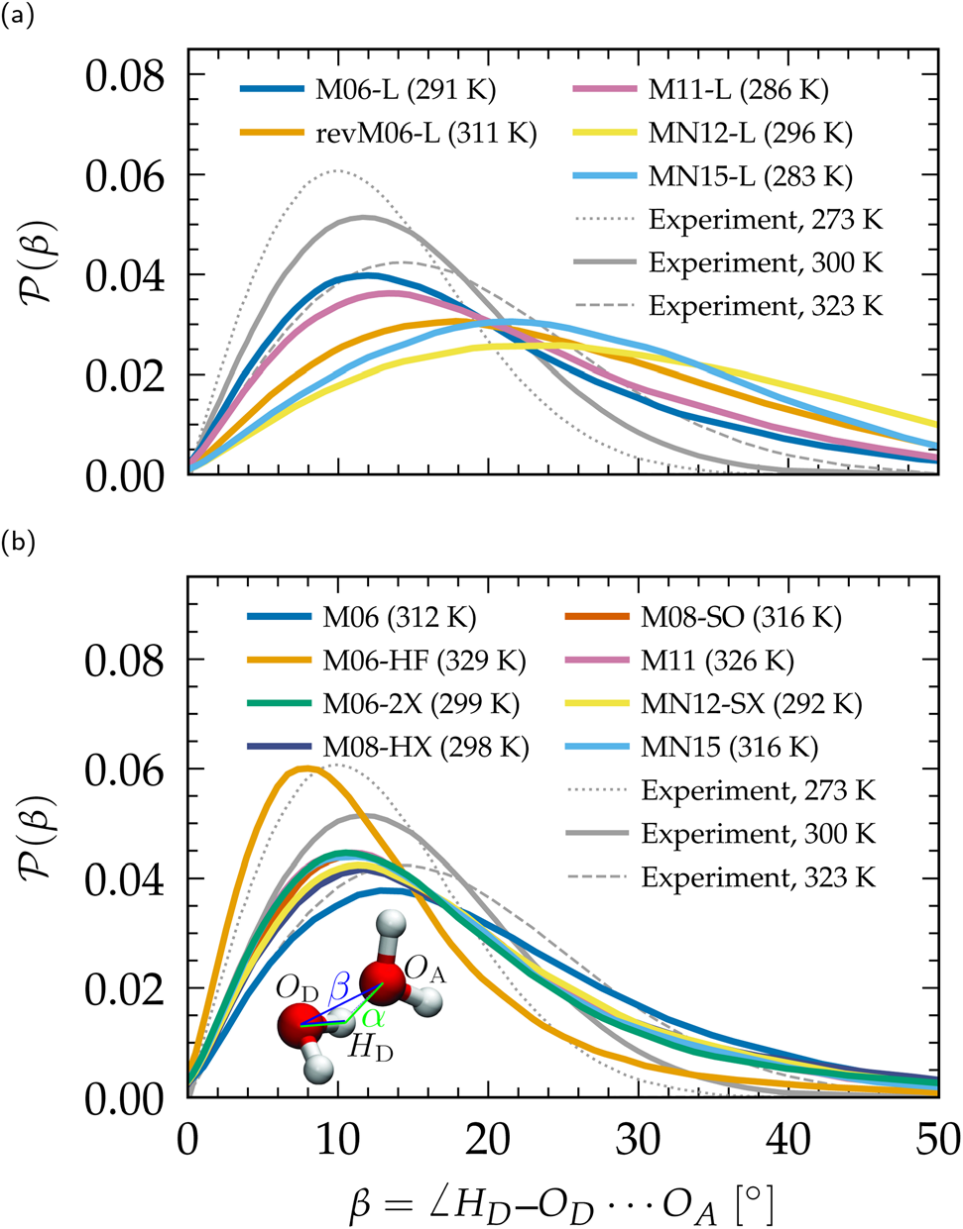
Distribution *P*(*β*) of the H-bonding angle *β*, compared to experimental values.^210^ (a) Meta-GGA Minnesota functionals, (b) hybrid meta-GGA Minnesota functionals. Distributions of the complementary angle *α* are provided in the ESI (Fig. S3†).

Linked to the fact that Minnesota meta-GGAs are not providing accurate descriptions of the structure of water (Fig. 1), being either understructured (M06-L, revM06-L, M11-L) or biasing the orientation between neighboring molecules (MN12-L, MN15-L), they are also unable to properly account for hydrogen bonds. Their angular distribution in Fig. 4a further shows that semilocal Minnesota functionals are incapable of capturing the full details of the hydrogen bond network of water, that is too fluid.

The hydrogen bond network of water is composed of a combination of short, straight, and robust bonds as well as longer, weak, and bent interactions. The strength of a hydrogen bond is consequently highly correlated with its length and angular orientation. At finite temperature, the elongation of the H-bonds competes with the cohesive effects of vdW interactions, which explains why the *h* number is in general higher and in better agreement with experimental data with dispersion corrections without altering significantly the angular distribution.^124^ In contrast, both *h* and the angular distribution vary when considering different fractions of exact exchange; Fig. 3c shows that *h* increases for M06-HF (100%), M06-2X (54%), M08-HX (52%), M08-SO (57%), while it is too low for M06 (27%), M11 (43–100%), MN12-SX (25–0%) and MN15 (44%). At the same time, H-bonds become shorter (Fig. 1, *g*_OH_) and straighter (Fig. 4b) when augmenting the fraction of exact exchange from M06 (27%) to M06-2X (54%) to M06-HF (100%). Hydrogen bonds are therefore particularly more sensitive to exchange effects than to correlation ones. Incorporating more exact exchange strengthens the hydrogen bonds and results in a more rigid structure of water.

Of all the structural properties analyzed, and taking also potential variations due to NQEs into account, we conclude that the functionals that provide results closest to experiments are: revPBE-D3, optB88-vdW, BLYP-DCACP (GGA), B97M-rV (meta-GGA), revPBE0-D3, PBE0-D3 (hybrid), M06-2X-D3, SCAN0 (hybrid meta-GGA) and the RPA (rung-5). Satisfactory agreement with experimental results, while directly accounting for NQEs, has only been demonstrated for revPBE0-D3 ^117,204^ and the RPA.^165^ The revPBE-D3,^204^ PBE0-D3 ^154^ and SCAN0 ^164^ functionals overstructure water with NQEs, while B97M-rV^117^ understructures. From a structural perspective, the remaining optB88-vdW and BLYP-DCACP GGAs emerge as intriguing candidates to investigate also in the presence of NQEs. The rung-4 M06-2X-D3 functional is even more promising, as it is slightly overstructured without NQEs and offers accurate density and hydrogen bond characteristics.

### 3.2 Dynamical properties

#### 3.2.1 Diffusion coefficient and orientational correlation times

**Fig. 5:**
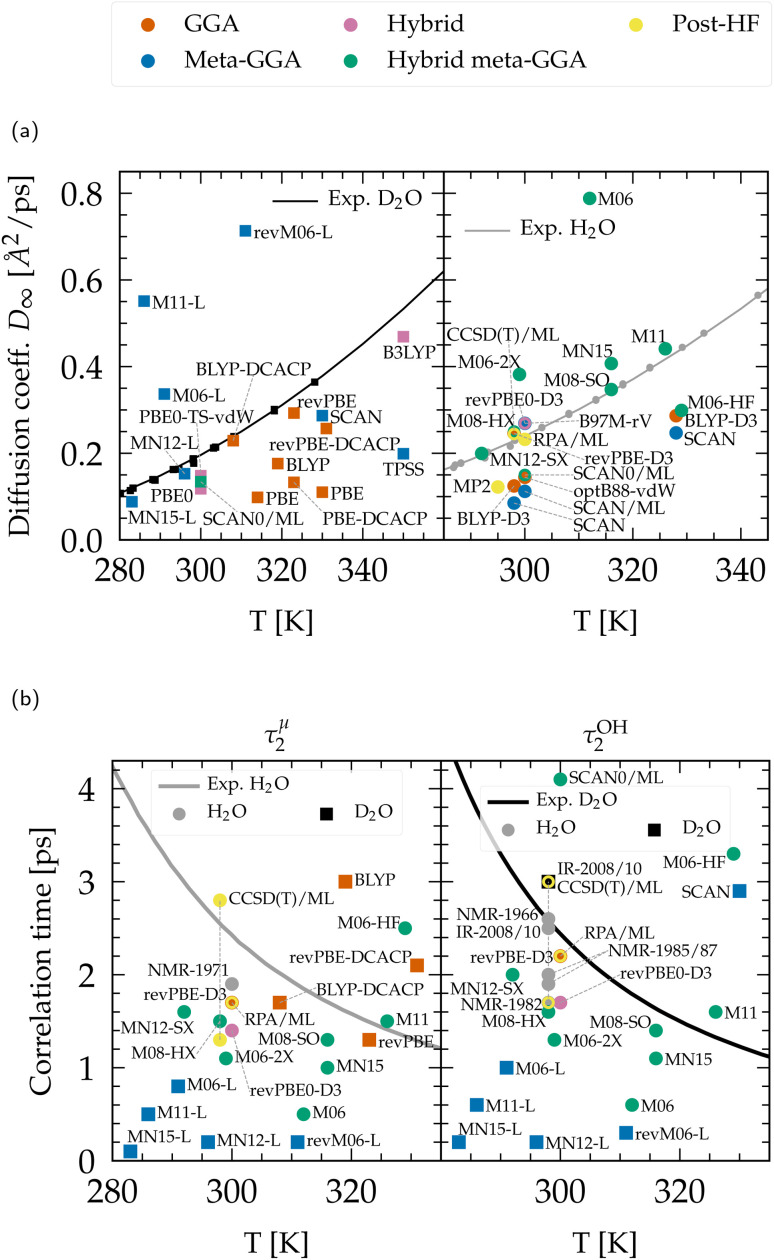
(a) Diffusion coefficient rescaled to infinite size for heavy (left) and light (right) water. Experimental data points were compiled from ref. 47–49, 51, 211 and 212 and fitted according to ref. 51. (b) Qualitative comparison of the orientational relaxation times *τ*^*μ*^_2_ and *τ*^OH^_2_ with experimental results.^42,46,50,52,198–201,213^ CCSD(T)/ML values are from PIMD simulations including NQEs,^197^ and extend through the range of experiments. Non-Minnesota results were extracted from ref. 40, 41, 117, 124, 140, 143, 154, 162–165, 184 and 204 and reported in Tables S4 and S5 of the ESI.†

In Fig. 3d, we plot the difference between the diffusion coefficient *D*_L_ and its experimental counterpart rescaled to a fictitious simulation box. The equivalent comparison with simulated coefficients *D*_∞_, rescaled to infinite size, is presented in Fig. 5a. While the diffusion coefficient provides information about the translational movement, rotational features are characterized by the orientational relaxation times plotted in Fig. 5b. These correlation times are highly sensitive to statistical sampling and require trajectories that are sufficiently long (approximately three times their value) to be accurately converged. Additionally, the fitting, respectively integration methods used for their calculation vary between studies, and experimental measurements exhibit non-negligible deviations. Nevertheless, these values are presented as a qualitative comparison.

The dynamics predicted by DFT functionals depends on their ability to account for hydrogen bond strength as well as directionality. Diffusion and rotational movements are determined by the dynamic breaking and formation of H-bonds under thermal fluctuations. Therefore, if the description of H-bonds is too strong, it significantly slows down the dynamical properties. Local and semilocal functionals suffer from the self-interaction error that promotes a delocalization of the protons.^143,164^ This delocalization facilitates the formation of H-bonds when the proton moves toward the acceptor and thus contributes to the H-bond strengthening, in an analogous manner to the NQEs (Section 3.1.2). As an illustration, the diffusion coefficient is too low for most GGA and meta-GGA functionals, in agreement with their tendency to overstructure. For example, optB88-vdW yields slightly overstructured water (Fig. 2), and diffuses too slowly (Fig. 5a). BLYP-DCACP and revPBE have higher diffusion coefficients, more in line with experiment, but this originates from their underestimation of the number of hydrogen bonds (Fig. 3c).

For GGAs, both BLYP-DCACP and revPBE-D3 functionals have a diffusion coefficient and relaxation times very close to the experiment, which is also true for the diffusion modelled by the B97M-rV meta-GGA. In contrast to the statement that the self-interaction error slows down the dynamics of liquid water, we have found that the M06-L, revM06-L and M11-L semilocal functionals exhibit a complete opposite trend, generally leading to faster dynamics. This is obviously due to their distortion of the hydrogen bonding network (Fig. 4) and incorrect structuring (Fig. 1). The diffusion coefficients predicted by MN12-L and MN15-L functionals appear to be in good agreement with experimental values (Fig. 5a), but this is fortuitously caused by an error compensation between the lack of hydrogen bonds (Fig. 3c) and their overly strong (incorrect) structure (Fig. 1). Their rotational dynamics is indeed significantly faster than observed experimentally (Fig. 5b).

As explained earlier, NQEs tend to strengthen H-bonds and slow down the dynamical properties. This was seen in all PIMD calculations with BLYP-D3,^184^ revPBE-D3,^204^ SCAN,^40,163,184^ B97M-rV,^117^ revPBE0-D3,^117,204^ and RPA/ML.^165^ The diffusion coefficients, in the absence of NQEs, should therefore be seen as overestimated, and relaxation times as underestimated. The diffusion with GGAs would therefore become even slower with NQEs. From the available data, hybrid and hybrid meta-GGA functionals generally give faster diffusion than GGAs, which indicates that the exact exchange is a key ingredient towards achieving accuracy for the dynamics, in the same way as for the structural properties. The revPBE0-D3 functional can be considered as the most effective hybrid in this regard. Other functionals like PBE0 or SCAN0 are likely to remain too slow even upon inclusion of NQEs.

Except for M06-HF, hybrid Minnesota functionals lead either to too fast diffusion or are in good agreement with the reference values. Thus, incorporating NQEs could potentially bring them closer to experimental results. Consistent with the understructuring tendency of M06 (with 27% of exact exchange) and the overstructuring of M06-HF (with 100%) (Fig. 1), the M06 family shows once again that the amount of exact exchange tightly regulates the precision of the functional: dynamical properties are too slow for M06-HF due to the shortening of stronger H-bonds, while M06 is too fast. The balanced M06-2X (54%) is giving results that are inbetween and therefore closer to experimental values. From a first estimation based on ML potentials, the dynamics of the rung-5 RPA description resembles closely the one revPBE-D3 and is thus highly consistent with the experimental data.

Overall, considering the possible influence of NQEs on the analyzed structural and dynamical properties, the functionals that most closely align with experiments are: revPBE-D3 and BLYP-DCACP (GGA), B97M-rV (meta-GGA), revPBE0-D3 (hybrid), M06-2X(-D3), SCAN0 (hybrid meta-GGA) and the RPA (rung-5). Satisfactory agreement for both structural and dynamical properties while accounting for NQEs has only been demonstrated with revPBE0-D3 ^117,204^ and RPA/ML.^165^ revPBE-D3 ^204^ and SCAN0/ML^164^ descriptions tend to overstructure water yielding too slow dynamics, even when accounting for NQEs, while B97M-rV^117^ understructures and slightly accelerates diffusion. Based on our extensive analysis, BLYP-DCACP^124^ (GGA) and M06-2X(-D3)^154^ (hybrid meta-GGA) functionals therefore emerge as promising competitors to revPBE0-D3 ^117,204^, and the RPA,^154,165^ and warrant further investigation with PIMD approaches.

## 4 Conclusions

Water is the most abundant substance on Earth, and its liquid properties are distinct from those of other fluids, posing a challenge for *in silico* simulations not only of condensed water but also of aqueous chemistry. In this work, we explored the performance of Minnesota meta-GGA and hybrid meta-GGA density functionals in describing the structure and dynamics of liquid water *via ab initio* molecular dynamics simulations. Contrary to the prevailing belief that local and semilocal functionals overstructure water, leading to underestimation of dynamical properties, the non-hybrid Minnesota meta-GGAs exhibit the opposite trend. M06-L, revM06-L, and M11-L lead to understructuring of water, while MN12-L and MN15-L lack cohesive effects, resulting in increased intermolecular distances. This behavior can be attributed to the weakening of the hydrogen bond network causing dynamical fingerprints that are far too fast. On the other hand, while most of the hybrid Minnesota functionals remain understructured (M06, M08-HX, M08-SO, M11, MN12-SX, MN15), their dynamical properties generally improve over those obtained with local and semilocal functionals (*e.g.*, M06-L, revM06-L, M11-L, MN12-L, MN15-L). The inclusion of exact exchange was identified as a key ingredient for the correct description of hydrogen bonds leading to improved structural and dynamical properties. In contrast, we found that an excessive amount of exact exchange (M06-HF) shortens and strengthens the hydrogen bonds between molecules, thus giving water properties that are too glassy.

M06-2X turns out to be the best Minnesota functional tested for liquid water and one of the best DFT functionals reported so far for this system overall. Its D3 dispersion corrected version shows very good agreement for structural properties. Describing the complete picture of water from small to larger clusters, to the condensed phase, is highly non-trivial with DFT, because functionals showing good performance in the gas phase do not necessarily perform well in the liquid phase and *vice versa*.^124,125^ Very encouragingly, M06-2X has also been identified as one of the most accurate functionals for relative energies of water hexamers^214^ and binding energies of 16-mers and 17-mers.^215^ Furthermore, from the thorough benchmark by Goerigk and Grimme, M06-2X-D3 was found to be the best among 23 hybrid functionals for general main group thermochemistry, kinetics, and noncovalent interactions.^112^

Previous studies considering explicit NQEs in water, have identified the hybrid revPBE0-D3, and the rung-5 RPA (EXX + RPA, RPA@PBE) with the help of machine learning potentials, as the only two approximations that agree closely with experiments so far. This therefore encourages the investigation of the performance of M06-2X(-D3) functionals with NQEs *via* path integral approaches. Although it is unfortunate that this involves drastic computational overheads, our work provides further evidence that both exact exchange and appropriate (non-local) correlation are essential for accurately describing water interactions. This, in turn, suggests that well-balanced XC functionals from higher rungs of the Jacob's ladder are required for simulating complex biological systems in water with predictive accuracy. In this regard, determining whether M06-2X(-D3) are indeed one of the best functionals would avoid the resort to the significantly more expensive fifth rung of the Jacob's ladder.

## Data availability

Data and analysis scripts are provided on Zenodo at https://doi.org/10.5281/zenodo.7933087.

## Author contributions

Conceptualization: J. V., M. P. B., U. R.; methodology: J. V., M. P. B., U. R.; software: J. V., M. P. B.; formal analysis: J. V., U. R.; investigation: J. V., M. P. B., U. R.; resources: U. R.; data curation: J. V.; writing – original draft preparation: J. V., U. R.; writing – review & editing: J. V., M. P. B., U. R.; visualization: J. V., M. P. B.; supervision: U. R.; project administration: J. V., U. R.; funding acquisition: U. R.

## Conflicts of interest

There are no conflicts to declare.

## Supplementary Material

SC-015-D3SC05828J-s001
