# Supplementary material for: Structure and dynamics of liquid water from ab initio simulations: adding Minnesota density functionals to Jacob's ladder
Source: Chem Sci. 2024 Feb 15;15(12):4434–51. doi: 10.1039/d3sc05828j (PMC10952088; doi:10.1039/d3sc05828j)
Supplement: SC-015-D3SC05828J-s001 [file SC-015-D3SC05828J-s001.pdf]

Electronic Supplementary Material (ESI) for Chemical Science.
This journal is © The Royal Society of Chemistry 2024

Structure and dynamics of liquid water from ab initio simulations: Adding Minnesota density functionals to Jacob's ladder

Justin Villard[a], Martin P. Bircher[b], and Ursula Rothlisberger*[a]

[a]*Laboratory of Computational Chemistry and Biochemistry, Institute of Chemical Sciences and Engineering, École Polytechnique Fédérale de Lausanne (EPFL), CH-1015 Lausanne, Switzerland*
[b]*Computational and Soft Matter Physics, Universität Wien, A-1090 Wien, Austria*

E-mail: ursula.roethlisberger@epfl.ch

Electronic supplementary information (ESI)

Simulation details

Table S1 Simulation details (production phase) for the different density functionals studied. M06-2X[a] corresponds to a similar simulation protocol conducted at larger wavefunction cutoff $E_{cut}^{\phi} = 120$ Ry, against 80 Ry for other trajectories. The BLYP GGA functional is shown for comparison.

Functional	N_{mol}	Dynamics	t_{traj} [ps]	n	Δt [a.u. $\mid$ fs]	$\bar{t}^{\mathrm{outer}}$ [min]	$\bar{t}^{\mathrm{inner}}$ [min]	Speedup	$\bar{t}_{\mathrm{sim}}$ [days/ps]	$(dE/dt)_{\max}$ [a.u./ps $\mid$ %]
					Meta-GGA					
M06-L	64 D_2O	CP	10.0	1	$2.0 \mid 0.048$	-	0.029	-	0.42	$0.000015 \mid 1 \cdot 10^{-6}$
revM06-L	64 D_2O	CP	10.0	1	$3.5 \mid 0.085$	-	0.035	-	0.28	$0.000003 \mid 3 \cdot 10^{-7}$
M11-L	64 D_2O	CP	10.2	1	$3.5 \mid 0.085$	-	0.037	-	0.30	$0.000919 \mid 8 \cdot 10^{-5}$
MN12-L	64 D_2O	CP	10.2	1	$3.5 \mid 0.085$	-	0.031	-	0.26	$0.000003 \mid 2 \cdot 10^{-7}$
MN15-L	64 D_2O	CP	10.2	1	$3.0 \mid 0.073$	-	0.027	-	0.26	$0.000009 \mid 9 \cdot 10^{-7}$
					Hybrid meta-GGA					
M06	32 H_2O	ML-MTS	6.0	6	$90.0 \mid 2.177$	88.82	0.08	5.97	30	$0.004870 \mid 9 \cdot 10^{-4}$
M06-HF	32 H_2O	ML-MTS	6.0	6	$90.0 \mid 2.177$	41.72	0.09	5.92	14	$0.003075 \mid 6 \cdot 10^{-4}$
M06-2X	32 H_2O	ML-MTS	7.1	10	$150.0 \mid 3.628$	21.75	0.10	9.57	4	$0.001272 \mid 2 \cdot 10^{-4}$
M06-2X[a]	32 H_2O	ML-MTS	7.0	10	$150.0 \mid 3.628$	42.83	0.19	9.58	7	$0.000823 \mid 1 \cdot 10^{-4}$
M08-HX	32 H_2O	ML-MTS	8.7	6	$90.0 \mid 2.177$	16.94	0.09	5.81	6	$0.000621 \mid 1 \cdot 10^{-4}$
M08-SO	32 H_2O	ML-MTS	6.8	10	$150.0 \mid 3.628$	21.36	0.10	9.57	4	$0.005239 \mid 1 \cdot 10^{-3}$
M11	32 H_2O	ML-MTS	6.0	10	$150.0 \mid 3.628$	87.87	0.16	9.83	16	$0.003982 \mid 7 \cdot 10^{-4}$
MN12-SX	32 H_2O	ML-MTS	6.0	6	$90.0 \mid 2.177$	18.91	0.13	5.77	6	$0.000833 \mid 2 \cdot 10^{-4}$
MN15	32 H_2O	ML-MTS	6.0	10	$150.0 \mid 3.628$	35.12	0.10	9.72	7	$0.007592 \mid 1 \cdot 10^{-3}$
					GGA					
BLYP	32 H_2O	BO	20.0	1	$15.0 \mid 0.363$	-	0.029	-	0.06	$0.003504 \mid 6 \cdot 10^{-4}$

N_{mol} is the number of heavy water molecules (D_2O), respectively light water (H_2O), simulated with meta-GGAs and hybrid meta-GGAs.

CP stands for Car-Parrinello dynamics while ML-MTS means Born-Oppenheimer (BO) dynamics accelerated with the machine-learning enhanced multiple time step scheme[119].

t_{traj} is the simulation length of the production phase. n is the ratio between inner and outer time steps when the ML-MTS scheme is used. $\Delta t = \delta t$ corresponds to the time step for CP dynamics, while for the ML-MTS scheme $\Delta t = n \cdot \delta t$ corresponds to the outer (physical) time step.

$\bar{t}^{\mathrm{outer/inner}}$ are the averaged elapsed times taken per outer/inner time step. We also report the ML-MTS speedup against standard BO dynamics from $\bar{t}^{\mathrm{outer}}$ and $\bar{t}^{\mathrm{inner}}$. $\bar{t}_{\mathrm{sim}}$ is the running time in order to get 1 ps of trajectory. Timings are reported for a full distribution of MPI tasks over 16 (13) Intel Xeon E5-2690 v3 @ 2.60GHz nodes with 12 cores each for respectively the meta-GGAs (hybrid meta-GGAs).

$(dE/dt)_{\max}$ represents the maximum energy fluctuation per time observed along each trajectory, in absolute and relative value compared to the average energy of the system.

Basis set convergence

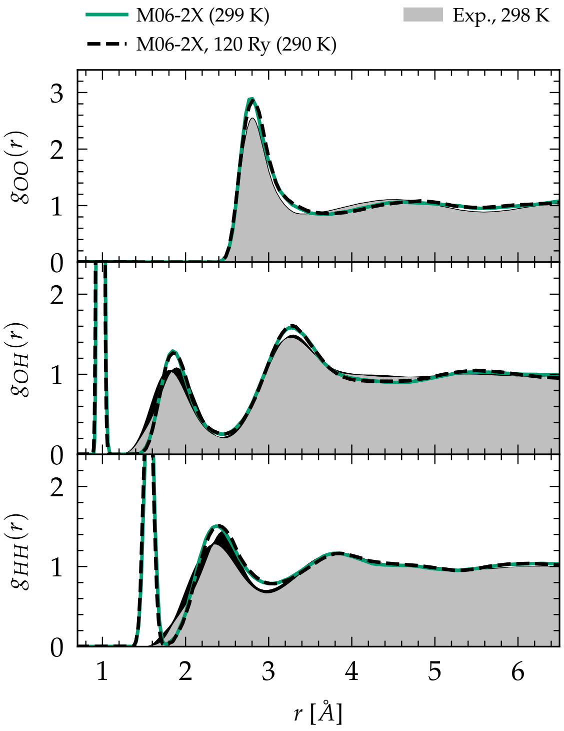

Fig. S1 Oxygen-oxygen (g_{OO}), oxygen-hydrogen (g_{OH}) and hydrogen-hydrogen (g_{HH}) radial distribution functions of liquid water predicted by the M06-2X Minnesota density functional, with a plane-wave wavefunction cutoff energy $E_{cut}^{\phi} = 120$ Ry (black), against 80 Ry (green). The experimental reference for g_{OO} comes from X-ray diffraction [54,55] interpolated at 298 K [197] and joint X-ray/neutron diffraction experiments were used for g_{OH} and g_{HH} [58]. Black areas represent experimental uncertainties.

Structural properties

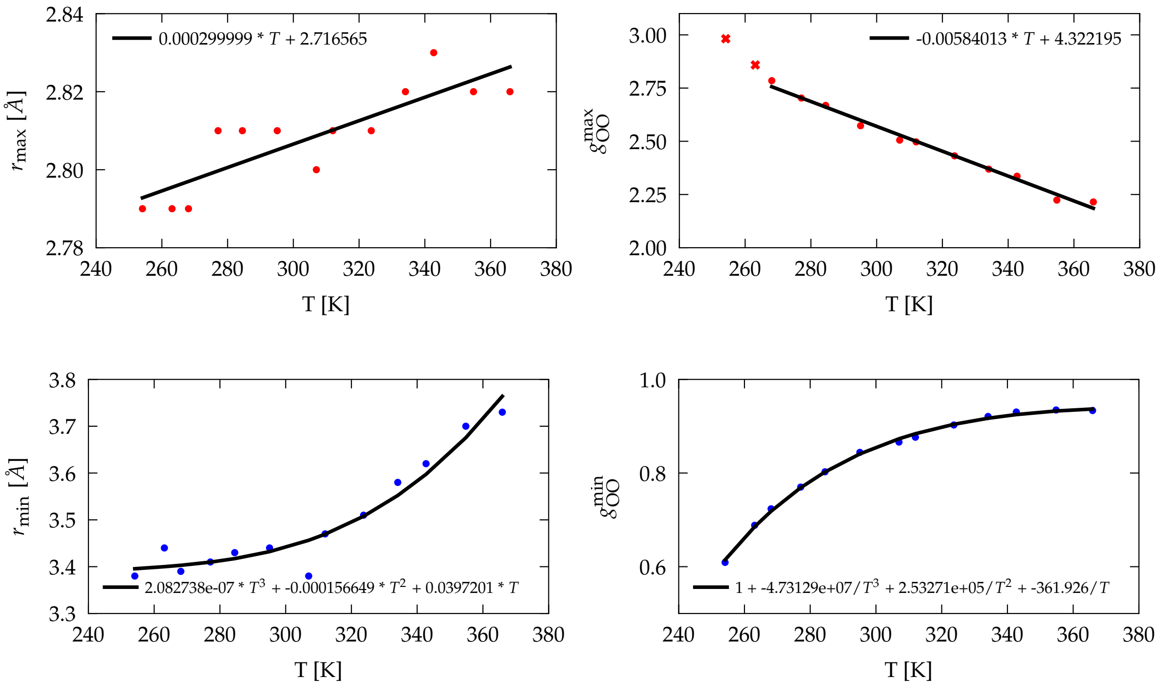

Fig. S2 Position $r_{\max}$ and height $g_{\mathrm{OO}}^{\max}$ of the first peak of the g_{OO} distribution at different temperatures extracted from X-ray measurements [55], as well as their first minimum analogues ($r_{\min}$, $g_{\mathrm{OO}}^{\min}$). Shown are the fitting curves used to rescale the simulated data to a common 298 K temperature, assuming a temperature dependency of DFT functionals similar to the experiment.

Table S2 Structure of liquid water. Position [Å] and height of the first maximum (r_{max}, g_{OO}^{max}) and first minimum (r_{min}, g_{OO}^{min}) of the oxygen-oxygen radial distribution function as obtained from MD or MC simulations with various DFT functionals at temperature T_{avg} [K]. Their normalized analogues (r^*_{max}, g_{OO}^{max*}) and (r^*_{min}, g_{OO}^{min*}) rescaled to 298 K were calculated from the experimental fits of Fig. S2. Corresponding references (cf. main text) are provided alongside the functional names.

			$T = T_{avg}$				$T = 298$ K		
Functional	T_{avg}	r_{max}	g_{OO}^{max}	r_{min}	g_{OO}^{min}	r^*_{max}	g_{OO}^{max*}	r^*_{min}	g_{OO}^{min*}
GGA									
BLYP [124]	319	2.77	2.86	3.31	0.66	2.76	2.98	3.26	0.61
BLYP-DCACP [124]	308	2.79	2.72	3.36	0.85	2.79	2.78	3.34	0.82
BLYP-D3 [154]	295	2.78	2.78	3.51	0.92	2.78	2.76	3.52	0.93
PBE [124]	314	2.72	3.19	3.27	0.43	2.72	3.28	3.23	0.39
PBE-DCACP [124]	323	2.71	3.27	3.28	0.40	2.70	3.42	3.21	0.35
PBE-D3 [154]	295	2.73	3.07	3.25	0.69	2.73	3.05	3.26	0.70
revPBE [124]	323	2.80	2.38	3.34	0.90	2.79	2.53	3.27	0.85
revPBE-DCACP [124]	331	2.74	2.94	3.35	0.76	2.73	3.13	3.25	0.70
revPBE-D3 [143]	298	2.81	2.59	3.52	0.89	2.81	2.59	3.52	0.89
rVV10 [154]	295	2.73	3.22	3.32	0.79	2.73	3.20	3.33	0.80
optB88-vdW [154]	295	2.74	2.94	3.34	0.80	2.74	2.92	3.35	0.81
Meta-GGA									
M06-L	291	2.85	2.36	4.50	0.92	2.85	2.32	4.51	0.94
revM06-L	311	3.09	2.37	4.58	0.72	3.09	2.45	4.55	0.69
M11-L	286	2.89	2.11	4.59	0.86	2.89	2.04	4.61	0.90
MN12-L	296	3.13	3.20	4.31	0.45	3.13	3.19	4.31	0.46
MN15-L	283	3.37	2.70	4.61	0.43	3.37	2.61	4.63	0.48
SCAN [40]	300	2.76	3.24	3.31	0.72	2.76	3.25	3.31	0.71
SCAN+rVV10 [138]	300	2.74	3.20	3.32	0.65	2.74	3.21	3.32	0.64
TPSS [140]	350	2.71	3.40	3.29	0.33	2.69	3.70	3.08	0.25
B97M-rV [117]	300	2.83	2.69	3.61	0.91	2.83	2.70	3.61	0.90
Hybrid									
B3LYP [140]	350	2.79	2.48	3.40	0.81	2.77	2.78	3.19	0.73
PBE0 [162]	300	2.71	2.96	3.30	0.53	2.71	2.97	3.30	0.52
PBE0-TS-vdW(SC) [162]	300	2.72	2.76	3.31	0.70	2.72	2.77	3.31	0.69
PBE0-D3 [154]	295	2.74	2.88	3.29	0.79	2.74	2.86	3.30	0.80
revPBE0-D3 [117]	300	2.80	2.57	3.47	0.89	2.80	2.58	3.47	0.88
Hybrid meta-GGA									
M06	312	2.85	2.24	4.70	0.91	2.85	2.32	4.67	0.88
M06-HF	329	2.65	2.72	3.22	0.63	2.64	2.90	3.13	0.57
M06-2X	299	2.81	2.89	3.74	0.85	2.81	2.90	3.74	0.85
M06-2X, $E_{cut}^{\phi} = 120$ Ry	290	2.81	2.86	3.71	0.86	2.81	2.81	3.72	0.89
M08-HX	298	2.82	2.96	4.02	0.81	2.82	2.96	4.02	0.81
M08-SO	316	2.85	3.01	4.10	0.91	2.84	3.12	4.06	0.87
M11	326	2.85	2.73	3.91	0.96	2.84	2.89	3.83	0.90
MN12-SX	292	2.93	2.91	3.86	0.86	2.93	2.87	3.87	0.88
MN15	316	2.85	2.30	4.47	0.87	2.84	2.41	4.43	0.83
M06-2X-D3 [154]	295	2.78	3.00	3.45	0.78	2.78	2.98	3.46	0.79
SCAN0/ML [164]	300	2.76	3.04	3.30	0.70	2.76	3.05	3.30	0.69
Post-HF, double-hybrid									
RPA [154]	295	2.78	2.93	3.41	0.78	2.78	2.91	3.42	0.79
RPA/ML [165]	300	2.79	2.89	3.41	0.83	2.79	2.90	3.41	0.82
MP2 [154]	295	2.76	3.05	3.32	0.72	2.76	3.03	3.33	0.73
PWPB95-D3 [154]	295	2.80	2.80	3.60	0.86	2.80	2.78	3.61	0.87
Experimental									
X-ray 2014 [55,197]	298	2.80	2.55	3.41	0.85	2.80	2.55	3.41	0.85

Table S3 Structure of liquid water. Coordination number $\bar{n}_{OO}$ calculated by integrating $g_{OO}(r)$ up to its first minimum. n_{OO} is the coordination number calculated by the same integration up to the first minimum of the radial distribution $4\pi r^2 g_{OO}(r)$ (eq 1 of the main text). Average number h of hydrogen bonds per water molecule and estimated equilibrium density ρ_{eq} [g/cm^3] relative to the experimental one ρ_{exp}[192] at same temperature. Results were obtained from MD or MC simulations with various DFT functionals at temperature T_{avg} [K]. Corresponding references (cf. main text) are provided alongside the functional names.

Functional	T_{avg}	$\bar{n}_{OO}$	n_{OO}	h	ρ_{eq}	ρ_{eq}/ρ_{exp}	System
GGA							
BLYP[124]	319	4.2	4.0	3.44	1.010	0.92	D_2O
BLYP-DCACP[124]	308	4.5	4.2	3.43	1.135	1.03	D_2O
BLYP-D3[154]	295	5.6	5.1	*3.66	1.066	1.07	H_2O
PBE[124]	314	4.0	4.0	3.58	1.056	0.96	D_2O
PBE-DCACP[124]	323	4.1	4.0	3.63	1.063	0.97	D_2O
PBE-D3[154]	295	4.3	4.0	*3.64	1.053	1.06	H_2O
revPBE[124]	323	4.2	3.8	3.20	0.931	0.85	D_2O
revPBE-DCACP[124]	331	4.7	4.3	3.59	1.114	1.02	D_2O
revPBE-D3[143]	298	5.6	4.6	*3.63	0.97	0.97	H_2O
rVV10[154]	295	4.6	4.2	*3.80	1.078	1.08	H_2O
optB88-vdW[154]	295	4.7	4.4	*3.84	1.081	1.08	H_2O
Meta-GGA							
M06-L	291	12.2	4.9	3.48	1.136	1.03	D_2O
revM06-L	311	12.8	9.9	2.90	1.171	1.06	D_2O
M11-L	286	12.9	6.9	3.22	1.171	1.06	D_2O
MN12-L	296	11.6	11.2	3.24	1.174	1.06	D_2O
MN15-L	283	13.4	13.0	1.93	1.280	1.16	D_2O
SCAN[41]	330	-	-	3.61	1.050	0.96	D_2O
SCAN[40]	300	4.7	4.4	-	-	-	H_2O
SCAN+rVV10[138]	300	4.6	4.4	*3.80	1.16	1.16	H_2O
TPSS[140]	350	-	-	3.82	-	-	D_2O
B97M-rV[117,143]	298	[117]5.8	[117]4.8	[117]*3.70	[143]1.12	1.12	[117,143]H_2O
Hybrid							
B3LYP[140]	350	4.4	4.0	3.67	-	-	D_2O
PBE0[154,162]	300	[162]4.1	[162]3.9	[162]3.71	[154]0.832	0.83	[162]D/[154]H_2O
PBE0-TS-vdW(SC)[162]	300	4.2	4.1	3.62	-	-	D_2O
PBE0-D3[154]	295	4.4	4.1	*3.68	1.053	1.06	H_2O
revPBE0-D3[117]	300	5.3	4.5	*3.80	-	-	H_2O
Hybrid meta-GGA							
M06	312	13.7	4.7	3.21	1.031	1.04	H_2O
M06-HF	329	3.9	3.9	3.55	1.051	1.07	H_2O
M06-2X	299	6.6	5.5	3.70	1.043	1.05	H_2O
M06-2X, $E^{\phi}_{cut} = 120$ Ry	290	6.6	5.5	3.71	-	-	H_2O
M08-HX	298	8.7	7.6	3.59	1.035	1.04	H_2O
M08-SO	316	9.0	5.0	3.70	1.033	1.04	H_2O
M11	326	7.6	4.4	3.46	1.074	1.09	H_2O
MN12-SX	292	7.8	6.8	3.36	1.025	1.03	H_2O
MN15	316	11.9	4.8	3.26	1.073	1.08	H_2O
M06-2X-D3[154]	295	5.1	4.7	*3.81	1.004	1.01	H_2O
SCAN0/ML[164]	300	4.5	4.2	3.71	1.032	1.04	H_2O
Post-HF, double-hybrid							
RPA[154]	295	4.7	4.2	*3.77	0.994	0.996	H_2O
MP2[154]	295	4.7	4.3	*3.81	1.020	1.022	H_2O
PWPB95-D3[154]	295	5.8	4.7	*3.62	1.002	1.004	H_2O
Experimental							
X-ray 2014[55]	285	4.8	4.5		0.99952	1.00	H_2O
X-ray 2014[55,197]/Neutron 2013[58]	298	[55,197]4.6	[55,197]4.3	[58]*3.80	0.99709	1.00	H_2O
X-ray 2014[55]	307	4.6	4.3		0.99442	1.00	H_2O
X-ray 2014[55]	324	5.2	4.5		0.98765	1.00	H_2O

*estimated from the integration of the second peak of the oxygen-hydrogen radial distribution function g_{OH}, in the same way as n_{OO}.

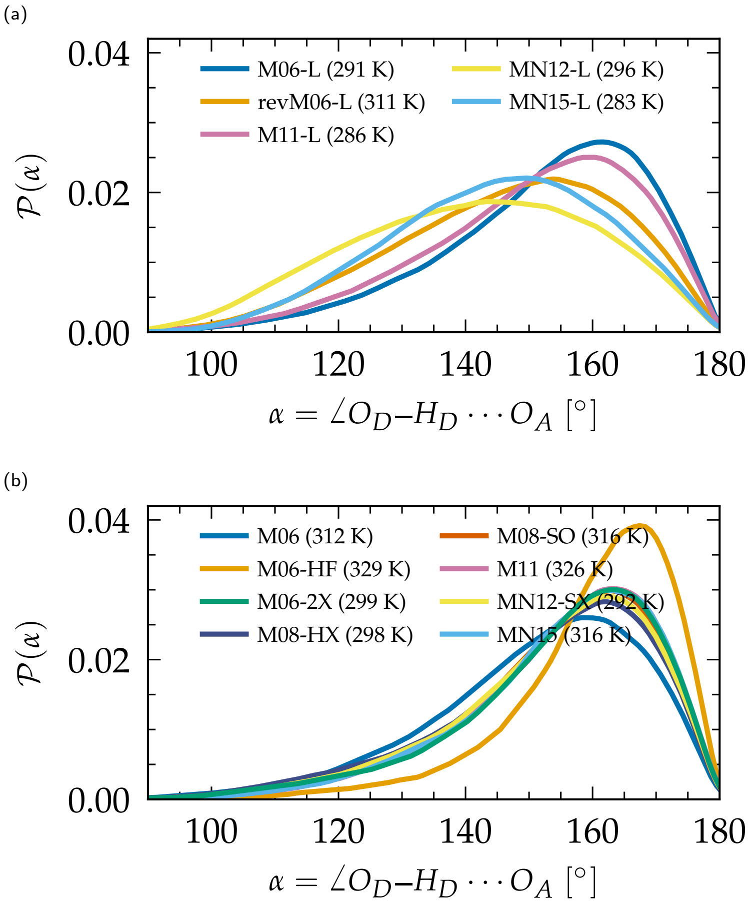

Fig. S3 Distribution $P(\alpha)$ of the H-bond donor angle α for donors in the first coordination shell. (a) Meta-GGA Minnesota functionals, (b) hybrid meta-GGA Minnesota functionals.

(a)

(b)

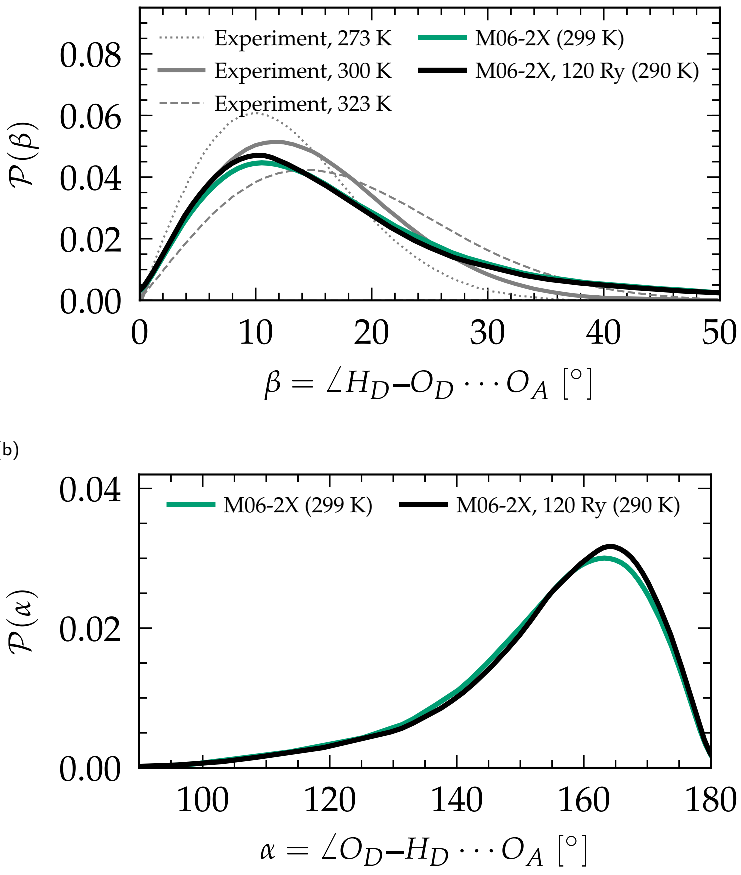

Fig. S4 H-bond angular distributions as predicted by the M06-2X Minnesota functional, with a plane-wave wavefunction cutoff energy $E^{\phi}_{cut} = 120$ Ry (black), against 80 Ry (green). (a) Distribution $P(\beta)$ of the H-bonding angle β, compared to experimental values[210]. (b) Distribution $P(\alpha)$ of the H-bond donor angle α for donors in the first coordination shell.

Dynamical properties

Table S4 Dynamics of liquid water. L [Å] is the side of the cubic simulation cell, D_L the finite-size diffusion coefficient from simulation and D_∞ its analogue rescaled to infinite size (eq 4 of the main text). Results were obtained from MD simulations with various DFT functionals at temperature $T_{\rm avg}$ [K]. Corresponding references (cf. main text) are provided alongside the functional names. $D_\infty^{\rm exp}$ is the experimental diffusion coefficient at $T_{\rm avg}$, as provided by a fractional-power law[51] fitted to experimental results[47–49,51,211,212]. $D_L^{\rm exp}$ is the experimental coefficient rescaled back to finite size (eq 4 of the main text). All diffusion coefficients are in [Å^2/ps]. η [mPa·s] is the experimental shear viscosity[192] of light/heavy water used for rescaling.

Functional	$T_{\rm avg}$	L	D_L	$D_L^{\rm exp}$	D_∞	$D_\infty^{\rm exp}$	$\eta(T_{\rm avg})$	System
				GGA				
BLYP[124]	319	12.420	0.10	0.23	0.18	0.30	0.70167	D_2O
BLYP-DCACP[124]	308	12.420	0.17	0.18	0.23	0.24	0.87277	D_2O
BLYP-D3[184]	298	15.640	0.08	0.18	0.12	0.23	0.88982	H_2O
BLYP-D3[184]	328	15.640	0.20	0.35	0.29	0.43	0.50354	H_2O
PBE[124]	314	12.420	0.03	0.21	0.10	0.27	0.77176	D_2O
PBE[41]	330	13.108	0.02	0.29	0.11	0.38	0.58027	D_2O
PBE-DCACP[124]	323	12.420	0.05	0.25	0.13	0.33	0.65294	D_2O
revPBE[124]	323	12.420	0.21	0.25	0.29	0.33	0.65294	D_2O
revPBE-DCACP[124]	331	12.420	0.16	0.29	0.26	0.39	0.57100	D_2O
revPBE-D3[143]	298	12.420	0.19	0.17	0.25	0.23	0.88982	H_2O
optB88-vdW[40]	300	9.850	0.07	0.17	0.14	0.24	0.85072	H_2O
				Meta-GGA				
M06-L	291	12.445	0.30	0.12	0.34	0.15	1.32310	D_2O
revM06-L	311	12.445	0.65	0.19	0.71	0.26	0.81967	D_2O
M11-L	286	12.445	0.52	0.10	0.55	0.13	1.53360	D_2O
MN12-L	296	12.445	0.11	0.13	0.15	0.18	1.15640	D_2O
MN15-L	283	12.445	0.06	0.09	0.09	0.12	1.68780	D_2O
SCAN[41]	330	12.217	0.19	0.28	0.29	0.38	0.58027	D_2O
SCAN[184]	328	12.660	0.14	0.32	0.25	0.43	0.50354	H_2O
SCAN[40]	300	9.850	0.06	0.17	*0.09	0.24	0.85072	H_2O
SCAN[184]	298	12.660	0.03	0.17	0.08	0.23	0.88982	H_2O
SCAN/ML[163]	300	11.817	0.05	0.18	0.11	0.24	0.85072	H_2O
TPSS[140]	350	9.939	0.03	0.36	0.20	0.53	0.43331	D_2O
B97M-rV[117]	300	12.420	0.21	0.18	0.27	0.24	0.85072	H_2O
				Hybrid				
B3LYP[140]	350	9.939	0.30	0.36	0.47	0.53	0.43331	D_2O
PBE0[162]	300	12.400	0.07	0.15	0.12	0.20	1.04660	D_2O
PBE0-TS-vdW(SC)[162]	300	12.400	0.10	0.15	0.15	0.20	1.04660	D_2O
revPBE0-D3[117]	300	12.420	0.21	0.18	0.27	0.24	0.85072	H_2O
				Hybrid meta-GGA				
M06	312	9.939	0.69	0.22	0.79	0.31	0.66506	H_2O
M06-HF	329	9.939	0.16	0.30	0.30	0.44	0.49563	H_2O
M06-2X	299	9.939	0.31	0.16	0.38	0.23	0.86991	H_2O
M06-2X, $E_{cut}^{\phi} = 120$ Ry	290	9.939	0.33	0.13	0.39	0.19	1.08400	H_2O
M08-HX	298	9.939	0.18	0.16	0.25	0.23	0.88982	H_2O
M08-SO	316	9.939	0.24	0.23	0.35	0.34	0.61743	H_2O
M11	326	9.939	0.31	0.29	0.44	0.42	0.52001	H_2O
MN12-SX	292	9.939	0.14	0.14	0.20	0.20	1.02640	H_2O
MN15	316	9.939	0.30	0.23	0.41	0.34	0.61743	H_2O
SCAN0/ML[164]	300	24.575	0.11	0.17	0.13	0.20	1.04660	D_2O
SCAN0/ML[164]	300	24.575	0.12	0.21	0.15	0.24	0.85072	H_2O
				Post-HF				
RPA/ML[165]	300	11.817	0.17	0.18	0.23	0.24	0.85072	H_2O
MP2[154]	295	12.335	0.07	0.16	0.12	0.21	0.95417	H_2O
CCSD(T)/ML PIMD[197]	298	15.660	0.20	0.18	0.24	0.23	0.88982	H_2O

*rescaled to infinite size with the actual viscosity obtained with the SCAN functional[40].

Table S5 Dynamics of liquid water. First-order τ_1 and second-order τ_2 orientational relaxation times [ps] calculated from the orientational auto-correlation function (eq 5 of the main text), between respectively the geometric dipoles μ, OH, and HH vectors. Results were obtained from MD simulations with various DFT functionals at temperature T_{avg} [K]. Corresponding references (cf. main text) are provided alongside the functional names. Note that $\tau_{1,2}$ are highly sensitive to statistical sampling and require trajectories that are sufficiently long (approximately three times higher than their value) to be accurately converged, in addition to a sufficient equilibration phase at the beginning of the NVE sampling. Additionally, the fitting or integration methods used for their calculation vary between studies, and experimental results exhibit non-negligible deviations. Nevertheless, we provide these values as a qualitative comparison (with ~10% of error tolerance for Minnesota functionals).

Functional	T_{avg}	τ_1^{μ}	τ_2^{μ}	τ_1^{OH}	τ_2^{OH}	τ_1^{HH}	τ_2^{HH}	System
GGA								
BLYP[124]	319	[a]7.5	[a]3.0	-	-	-	-	D_2O
BLYP-DCACP[124]	308	[a]3.6	[a]1.7	-	-	-	-	D_2O
PBE[124]	314	[a]36.9	[a]15.6	-	-	-	-	D_2O
PBE[41]	330	-	-	-	[b]7.1	-	-	D_2O
PBE-DCACP[124]	323	[a]32.7	[a]10.0	-	-	-	-	D_2O
revPBE[124]	323	[a]2.7	[a]1.3	-	-	-	-	D_2O
revPBE-DCACP[124]	331	[a]5.4	[a]2.1	-	-	-	-	D_2O
revPBE-D3[204]	300	[b]4.6	[b]1.7	[b]5.4	[b]2.2	[b]5.9	[b]2.6	H_2O
Meta-GGA								
M06-L	291	1.8	0.8	2.3	1.0	2.7	1.3	D_2O
revM06-L	311	0.4	0.2	0.5	0.3	0.6	0.3	D_2O
M11-L	286	1.0	0.5	1.3	0.6	1.4	0.7	D_2O
MN12-L	296	0.4	0.2	0.5	0.2	0.5	0.3	D_2O
MN15-L	283	0.4	0.1	0.4	0.2	0.4	0.2	D_2O
SCAN[41]	330	-	-	-	[b]2.9	-	-	D_2O
SCAN/ML[163]	300	-	[b]12.9	-	[b]15.7	-	[b]21.5	H_2O
Hybrid								
revPBE0-D3[204]	300	[b]3.4	[b]1.4	[b]4.3	[b]1.7	[b]4.8	[b]2.0	H_2O
Hybrid meta-GGA								
M06	312	1.2	0.5	1.2	0.6	1.2	0.6	H_2O
M06-HF	329	4.7	2.5	6.2	3.3	7.3	3.6	H_2O
M06-2X	299	2.7	1.1	3.0	1.3	3.2	1.4	H_2O
M06-2X, $E_{cut}^{\phi} = 120$ Ry	290	2.6	1.2	3.3	1.5	3.8	1.7	H_2O
M08-HX	298	2.8	1.5	3.0	1.6	3.2	1.8	H_2O
M08-SO	316	2.8	1.3	3.0	1.4	3.2	1.6	H_2O
M11	326	3.0	1.5	3.6	1.6	3.9	1.9	H_2O
MN12-SX	292	3.7	1.6	4.4	2.0	4.9	2.9	H_2O
MN15	316	2.0	1.0	2.0	1.1	2.0	1.3	H_2O
SCAN0/ML[164]	300	-	-	-	[b]4.6	-	-	D_2O
SCAN0/ML[164]	300	-	-	-	[b]4.1	-	-	H_2O
Post-HF								
RPA/ML[165]	300	-	[b]1.7	-	[b]2.2	-	[b]2.6	H_2O
CCSD(T)/ML PIMD[197]	298	-	[b]1.3	-	[b]1.7	-	[b]2.1	H_2O
CCSD(T)/ML PIMD[197]	298	-	2.8	-	3.0	-	3.3	H_2O
Experimental								
NMR 1970[2,199]	300	4.8	-	-	-	-	-	H_2O
NMR 1971[199,213]	300	-	1.9	-	-	-	-	H_2O
NMR 1967 (for various T)[42]	300	-	2.4	-	-	-	-	H_2O
Infrared 2008[200]/2010[201]	298	-	-	-	2.5	-	2.5	H_2O
Infrared 2008[200]/2010[201]	298	-	-	-	3.0	-	-	D_2O
NMR 2001 (for various T)[52]	300	-	-	-	2.4	-	-	D_2O
NMR 1985[45]/1987[46]	298	-	-	-	1.9-2.0	-	-	H_2O
NMR 1982[50]	298	-	-	-	1.7	-	-	H_2O
NMR 1966[198]	298	-	-	-	2.6	-	-	H_2O
NMR 1976[43]	303	-	-	-	-	-	2.1	H_2O
NMR 1976[43]	303	-	-	-	-	-	2.5	D_2O

[a] $\tau_{1,2}$ were calculated from fitting the auto-correlation functions $C_{1,2}(t)$ with the exponential form $A\exp[-(t/\tau_{1,2})^{\alpha}]$.

[b] The tail of the auto-correlation function was fitted with $\exp(-t/\tau_{1,2})$ and integrated from zero to ∞ to give $\tau_{1,2}$.

Others were obtained with the fit $A\exp(-t/\tau_{1,2})$ in the exponential regime after the initial subpicosecond librational decay.
